# A comprehensive pan-cancer analysis of LRFN4: its potential as a prognostic biomarker and therapeutic target for immunotherapy

**DOI:** 10.3389/fimmu.2025.1539076

**Published:** 2025-05-02

**Authors:** Yinmei Xu, Xin Wu, Peng Zhi, Genyu Guo, Yankan Fu, Lukuan You, Siyuan Huai, Jianxiong Li

**Affiliations:** ^1^ Medical College, People’s Liberation Army General Hospital, Beijing, China; ^2^ Department of General Surgery, People’s Liberation Army General Hospital, Beijing, China; ^3^ Department of Radiotherapy, Fifth Medical Center of the People’s Liberation Army General Hospital, Beijing, China

**Keywords:** LRFN4, pan-cancer analysis, TCGA, immunotherapy, tumor microenvironment, prognostic biomarker

## Abstract

**Background:**

LRFN4, characterized by leucine-rich repeats and fibronectin type III domains, has been implicated in various human diseases. However, its role in immune regulation and cancer prognosis remains unclear.

**Methods:**

We performed a comprehensive analysis using datasets from The Cancer Genome Atlas (TCGA), Cancer Cell Line Encyclopedia (CCLE), Genotype-Tissue Expression Project (GTE x), UALCAN, Star Base, and Comparative Toxicogenomics Database (CTD), and observed significant dysregulation of LRFN4 in multiple cancers compared to normal tissues.

**Results:**

LRFN4 expression was strongly correlated with clinical prognosis, immune subtypes, molecular subtypes, immune checkpoint (ICP) genes, tumor mutational burden (TMB), microsatellite instability (MSI), and immune infiltration, which were measured by ESTIMATE scores. Moreover, LRFN4 expression was associated with the presence of tumor-infiltrating immune cells, particularly in gastrointestinal tumors, reflecting immune cell genetic signatures. Validation through fluorescence multiplex immunohistochemistry confirmed that the association of LRFN4 protein expression with the clinicopathological features and the immune microenvironment of gastric cancer. Flow cytometry analysis indicated that LRFN4 inhibited apoptosis in gastric cancer cell lines while enhancing cell cycle arrest in the S phase. Western Blot analysis demonstrated a positive correlation between the high expression of LRFN4 and the expression levels of cyclin D1 as well as CDK4. In contrast, a negative correlation was observed between the high expression of LRFN4 and the expression level with cleaved-caspase-3 levels.

**Conclusion:**

These findings suggest that LRFN4 may serve as a novel biomarker for cancer prognosis and a potential target for immunotherapy.

## 1 Introduction

Cancer remains a major global public health challenge, with its complexity stemming from diverse tumorigenic processes and molecular mechanisms. Conducting pan-cancer analyses of tumor-related genes is critical for understanding their roles in clinical prognosis and identifying the molecular pathways driving oncogenesis. Such analyses establish a robust foundation for the discovery of therapeutic targets and the development of novel treatment strategies (1–3). Recent advances in immunotherapy, particularly with immune checkpoint inhibitors targeting PD-1/PD-L1 and CTLA-4, have transformed the management of advanced cancers (4, 5). Despite these successes, the tumor microenvironment (TME) poses substantial challenges, mediating resistance and variability in treatment efficacy. The TME consists of a complex interplay between tumor cells, immune cells, stromal components, and soluble factors, such as cytokines and chemokines. Tumor cells manipulate the functions of immune and stromal cells by the secretion of growth factors and cytokines, creating an environment favorable to tumor progression and immune evasion. These interactions highlight the pressing need to identify novel prognostic biomarkers to enhance the precision and efficacy of immunotherapy (6).

LRFN4 protein, also known as leucine-rich repeat and fibronectin type III domain-containing protein 4, is distinguished by the presence of leucine-rich repeats and a fibronectin type III domain (7, 8). It plays a significant role in various cellular processes, including cell proliferation, cell cycle regulation, and the modulation of cellular inflammation. Numerous studies have investigated LRFN4, establishing a functional connection between LRFN4 and the development of tumors, including in colon cancer, lung cancer, and leukemia. For example, in colon cancer, LRFN4 has been shown to promote cell proliferation through regulating specific signaling pathways. In lung cancer, its overexpression has been associated with enhanced cell invasion ability. In leukemia, LRFN4 might contribute to tumorigenesis by disrupting the normal regulation of hematopoiesis (9–11). This study presents a comprehensive summary of current evidence derived from cellular and animal experiments, highlighting the association between LRFN4 and different types of cancer.

This study represents the first pan-cancer analysis of LRFN4 utilizing data from the TCGA project and the GEO database. Additionally, we integrate a broad array of investigations, including gene expression, survival status, DNA methylation, genetic alterations, protein phosphorylation, immune infiltration, and associated cellular pathways, to elucidate the potential molecular mechanisms through which LRFN4 may influence the onset or clinical prognosis of various cancers.

## 2 Materials and methods

### 2.1 Gene expression analysis

RNA-sequencing (RNA-seq) data for 33 tumor types were downloaded from the Cancer Genome Atlas (TCGA) database (12), which provides a comprehensive functional genomics data set encompassing various tumors, facilitating robust pan-cancer analyses. The STAR alignment pipeline was used to process the data, and transcripts per million (TPM) expression values were extracted. Data analysis and visualization were conducted using R software (version 4.2.1). Statistical tests based on data characteristics were performed using the stats (version 4.2.1) and car (version 3.1-0) packages. Analyses not meeting statistical requirements were excluded (13). The Wilcoxon rank sum test was used for group comparisons application. The expression profile of LRFN4 in cancer cell lines was analyzed using the Cancer Cell Line Encyclopedia (CCLE) database.

For tumors lacking normal tissue data (e.g., TCGA-GBM, TCGA-LAML), GEPIA2 was employed to compare TCGA tumor samples with GTEx normal tissues, using a *P*-value cutoff of 0.01 and a log_2_FC threshold of 1 (14, 15). The correlation between LRFN4 expression-pathological staging was assessed using GEPIA2’s “Pathological Staging Chart,” generating normalized (log_2_ [TPM + 1]) violin plots for stages I-IV. GEPIA2 was utilized for TCGA-GTEx data integration, with figures based on log-transformed values [log_2_(TPM + 1)].

### 2.2 Survival prognosis analysis

ROC analysis with the pROC package was used to evaluate LRFN4’s ability to distinguish tumors from normal tissues. Data from UCSC XENA, where TCGA and GTEx datasets were processed into TPM format via the Toil pipeline (16, 17). Prognostic Analysis, RNA-seq and clinical data from TCGA exploring overall survival (OS) and disease-free survival (DFS). GEPIA2 provided OS and DFS maps, stratifying cohorts into high- and low-expression groups (14). To further validate our findings, supplementary survival analysis was conducted using the Kaplan-Meier plotting tool, with data sourced from the GEO database. This analysis aimed to assess the prognostic significance of LRFN4 expression across multiple cancer types.

### 2.3 Immune and molecular subtype analysis

The TISIDB portal (http://cis.hku.hk/TISIDB/, accessed on September 3, 2024) was utilized to investigate the relationship between LRFN4 expression and the immune or molecular subtypes of various cancers.

### 2.4 Biomarker efficacy analysis

The TCGA Pan-Cancer dataset (PANCAN, N = 10,535, G = 60,499) was used to investigate the relationship between LRFN4 expression and key therapeutic biomarkers, including tumor mutational burden (TMB), microsatellite instability (MSI), and the ESTIMATE scores. Expression profiles for the gene ENSG00000173621 (LRFN4) were extracted, with samples were filtered to include only “Primary Blood Derived Cancer - Peripheral Blood” and “Primary Tumor” types. Cancer types with fewer than three samples were excluded, resulting in data for 37 cancer types. TMB was calculated using MuTect2-processed simple nucleotide variation data, and the “maftools” R package (18–20). MSI scores were retrieved from TCGA’s public data. Tumor microenvironment characteristics (ESTIMATE scores) were obtained through the ESTIMATE algorithm (21, 22). All expression data were normalized using a log_2_(x + 0.001) transformation before integration.

### 2.5 Genetic alteration analysis

The cBioPortal platform (https://www.cbioportal.org/, accessed on September 3, 2024) was used to investigate LRFN4’s genetic alterations in a pan-cancer cohort. We selected the “TCGA Pan Cancer Atlas Studies” dataset to characterize mutation frequencies, mutation types, and copy number alterations using the “Cancer Types Summary” module. The Simple Nucleotide Variation Level 4 dataset from the GDC portal, which was processed by MuTect2, was used. Mutation data were annotated with protein domain information using the “maftools” R package (version 2.2.10) to map mutations to specific protein domains (19, 20). Mutation hotspots within critical functional domains were identified, highlighting potential targets for further experimental validation.

### 2.6 Immune gene correlation analysis

A correlation analysis was performed to investigate the relationship between LRFN4 expression and immune checkpoint genes using the standardized pan-cancer dataset from the UCSC database (https://xenabrowser.net, accessed on September 3, 2024). Specifically, we utilized the TCGA TARGET GTEx dataset (PANCAN, N=19,131, G=60,499) (17). The gene ENSG00000173621 (LRFN4) along with 60 immune checkpoint-related genes, including 24 inhibitory and 36 stimulatory genes, as defined in The Immune Landscape of Cancer (23), were extracted. We focused on samples derived from primary solid tumors and primary hematologic malignancies (bone marrow and peripheral blood), excluding normal tissue to ensure a tumor-specific context. Expression values were log_2_(x+0.001)-transformed to stabilize variance and improve data normalization. Pearson correlation coefficients were calculated to quantify the relationship between LRFN4 and immune checkpoint genes. We further applied this methodology to analyze the correlation between LRFN4 and five immune pathway marker gene sets. The following selects eight widely - studied immune checkpoint - related genes to specifically analyze their correlations with LRFN4.

### 2.7 Immune infiltration analysis

The xCell method (23), as implemented through the IOBR R package (24) (version 0.99.9), is employed to deconvolute gene expression data into immune infiltration scores for 67 immune and stromal cell types, thereby assessing immune cell infiltration in tumor samples.In addition, we utilized the “immune-gene” module of the TIMER2 web server (http://timer.cistrome.org/, accessed on September 4, 2024) to investigate the association between LRFN4 expression and immune cell infiltration across TCGA tumor types, with a focus on key cell subsets such as CD8+ T cells and cancer-associated fibroblasts (CAFs) (25). We focused our analysis on seven gastrointestinal cancers (esophageal, gastric, colorectal, liver, biliary tract, pancreatic, and small intestine cancers) due to their high incidence and mortality rates, as well as the availability of comprehensive data from public databases such as GEO and TCGA. These cancers share common features in the tumor microenvironment and immune response, making them ideal for investigating the role of LRFN4 in immune cell infiltration and tumor progression.Key immune cell subsets such as CD8+ T cells and cancer-associated fibroblasts (CAFs) (25) play a critical role in shaping the immunosuppressive TME. Immune infiltration was quantified using multiple computational algorithms, including TIMERCIBERSORT (25), CIBERSORT-abs, QUANTISEQ (26), xCell (23), MCPCOUNTER (27), and EPIC (28). This multi-algorithm approach ensured a comprehensive assessment of immune infiltration. Spearman rank correlation tests, adjusted for tumor purity, were applied to obtain *P*-values and partial correlation coefficients (cor).

### 2.8 Enrichment analysis

PPI analysis of LRFN4 (Homo sapiens) was conducted using the STRING database (https://string-db.org, accessed on September 4, 2024) (28), with parameters set to an interaction score ≥ 0.150 (low confidence), and incorporating evidence-based edges, 50 first-shell interactors, and experimental validation as sources. The top 100 genes most strongly correlated with LRFN4 across TCGA datasets were identified using the GEPIA2 web tool (http://gepia2.cancer-pku.cn, accessed on September 4, 2024) (14) via the “similar gene detection” module. Pearson correlation (log_2_ TPM values) between LRFN4 and these genes was computed. Heatmaps showing partial Spearman correlation coefficients and adjusted p-values for tumor purity were generated by the “Gene_Corr” module of TIMER2 (http://timer.cistrome.org, accessed on September 4, 2024) (29). Overlapping genes between LRFN4-binding proteins and LRFN4-correlated genes were determined using Jvenn (https://bioinfo.genotoul.fr/jvenn, accessed on September 4, 2024), and subjected to KEGG pathway enrichment analysis (30–32), and enriched terms visualized using a cnetplot (circular = F, colorEdge = T, node_label = T).

All analyses were executed in R 3.6.3 (64-bit), considering *P* < 0.05 as statistically significant.

### 2.9 Cell culture

Human gastric cancer cell lines, HGC-27 and MKN-45, were procured from QINGQI (Shanghai) Biotechnology Development Co., Ltd. The cells were cultured in 1640 medium (SH30027.01, HyClone, China) supplemented with 10% fetal bovine serum (SH30396.02, HyClone, China) and 1% penicillin-streptomycin mixture (SV30010, HyClone, China). The cultivation occurred at 37°C in a 5% CO_2_ incubator (Jiemei Electronics, CI-191C). The cell culture medium was refreshed every two days, and the cells were employed for subsequent experiments upon reaching the exponential growth phase.

### 2.10 Construction of gastric cancer cell models

MKN-45 cells in the logarithmic growth phase were seeded into culture vessels until 50% - 70% confluence was achieved. Subsequently, an overexpressed lentivirus at an MOI of 20 was introduced for cell infection. After 48 hours, the cells were cultured in puromycin-containing medium for an additional 48 hours to select for successfully transfected cells, with the survival of virus-infected cells after puromycin treatment indicating successful transfection. The same procedure was performed for LRFN4 overexpression in the HGC-27 cells. For gene knockdown, an shRNA sequence targeting LRFN4 (shRNA1212: CCATAACCTTATTGACGCACT) was designed and integrated into a lentiviral vector. Subsequent experiments involved three groups for MKN-45 cells: Control, OE-NC, and OE-LRFN4, and for HGC-27 cells, the groups were Control, shNC, and shLRFN4.

The efficiency of LRFN4 knockdown and overexpression models was assessed through quantitative polymerase chain reaction (qPCR) and Western blot (WB) analyses. RNA was extracted post-treatment, reverse transcribed to cDNA and subjected to qPCR using LRFN4-specific primers with GAPDH as a reference. The ΔΔCt method determined relative mRNA levels. Protein lysates from treated cells were separated by SDS-PAGE, transferred to membranes, and probed with LRFN4 and GAPDH antibodies. Densitometry quantified LRFN4 protein expression relative to GAPDH.

### 2.11 Flow cytometry analysis for cell apoptosis and cell cycle detection

Cell lines with stable LRFN4 overexpression, LRNF4 knockdown, and corresponding controls were cultured separately at 37°C under 5% CO_2_ until suitable density was achieved. For apoptosis analysis, the culture medium was removed, and cells were washed twice with PBS to eliminate residues. Trypsin was added for cell detachment, and the resulting suspension was centrifuged at 1000g for 5 minutes. The supernatant was discarded, and the cell pellet was resuspended in PBS to a concentration of 1×10^6^ cells/ml. A 100 μl aliquot of the suspension was transferred to a flow tube, mixed with 5 μl of Annexin V-FITC and 5 μl of PI staining solution, and incubated at RT in the dark for 20 minutes. After adding 400μl of PBS, flow cytometry was used to detect and record the proportion of Annexin V-FITC and PI positive cells. Flow cytometer software distinguished early apoptotic (Annexin V^+^/PI^-^), late apoptotic (Annexin V^+^/PI^+^), and normal cells (Annexin V^-^/PI^-^). The apoptosis proportions of different groups were compared to assess the impact of LRFN4 manipulation.

**Figure 1:**
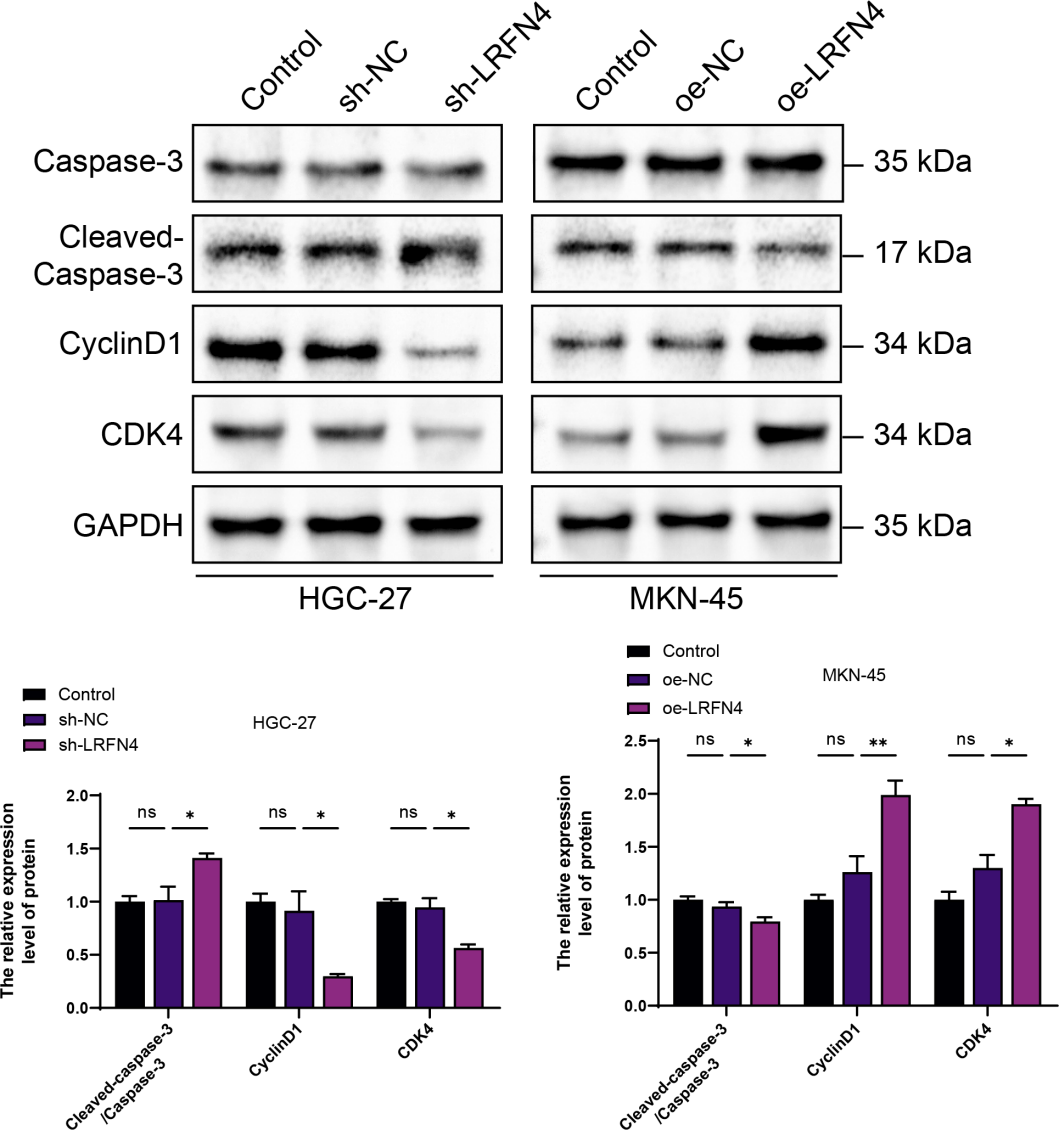
Elucidating the expression interrelationship between LRFN4 and apoptotic as well as cell-cycle-associated proteins in gastric cancer cell lines HGC-27 and MKN-45 using western blot (WB) analysis. NS, Not Significant; *P < 0.05, **P < 0.01.

For cell cycle analysis, exponentially growing cell lines (LRFN4 overexpression, knockdown, and control) were processed similarly. After cell pellet collection, pre-cooled 70% ethanol was added, and cells were gently mixed by pipetting and fixed at 4°C overnight. Cells were centrifuged at 1200g for 3 minutes to remove ethanol, washed twice with PBS, incubated with PI dye solution (containing RNase A) at RT in the dark for 30 minutes. The stained cell suspension was filtered into a loading tube for flow cytometry detection and fluorescence signal recording. The software was used to analyze cell cycle phase distribution (G0/G1, S, G2/M) (**Figure 1**).

### 2.12 Fluorescence-based mIHC analysis

Gastric cancer patient tissue arrays were sourced from Beijing Mescape Biotechnology Co., Ltd. The submitted samples were preserved in 4% paraformaldehyde. Following fixation, these specimens underwent meticulously trimmed, dehydrated, embedded, sectioned, stained, and sealed, strictly adhering to the standard operating procedures (SOP) of the pathology laboratory of the unit for detailed examination. The expression and localization of the target gene LRFN4 were analyzed using Visiopharm Intelligent Full-line AI Digital Pathology Quantitative Analysis Software. Additionally, SPSS was employed to analyze the relationship between LRFN4 gene expression and patient demographic variables such as gender, age, and clinical stage.

### 2.13 Western blot analysis

Cells were harvested at the logarithmic growth phase. The procedure involved washing with PBS, lysing with RIPA buffer on ice (30 min), scraping, centrifugation (12,000 rpm, 4°C, 5 min). Subsequent steps included an additional centrifugation (5000 rpm, 4°C, 5 min), lysis with RIPA (ice, 20–30 min), sonication, and further centrifugation (12,000 rpm, 4°C, 10 min). Protein concentrations were determined using a BCA kit with BSA standards, samples were mixed with reagent and incubate at 37°C (30 min), followed by absorbance measurement at 562nm. Samples were then mixed with 5× loading buffer, boiled, and centrifuged. Prepare gels, load samples, electrophorese (80 V to 120 V), transfer proteins to a 0.2 μm PVDF membrane (100 V, 1 h). Blocking was performed with 5% non-fat milk in PBS-T (room temperature, 1 h/4°C overnight), followed by incubation with appropriately diluted primary and secondary antibodies, and washing with PBS-T. Mix ECL solutions A and B, applied to the membrane (1 min), wrap, expose film (1-5min), develop, and fix. In the Western blot analysis, we used Abcam antibodies for target protein detection. The Cleaved-Caspase-3 (ab2303, 1:500), Caspase 3 (ab90437, 1:1000), Cyclin D1 (ab226977, 1:500), Cdk4 (ab108357, 1:1000), anti - LRFN4 (ab106369, 1:1000), and anti - GAPDH (ab8245, 1:3000) antibodies were applied.

### 2.14 Statistical evaluation

The data are expressed as mean values ± standard deviation (SD), with all experiments conducted in triplicate. All statistical evaluations were carried out using GraphPad Prism 7.0, SPSS (version 22.0), or R software (version 4.1.2). A significance threshold was set at *P* < 0.05. The following notation indicates statistical significance: ns for not significant; *P < 0.05; **P < 0.01; ***P < 0.001.

## 3 Results

### 3.1 Differential expression of LRFN4 in pan-cancer

**Figure 2:**
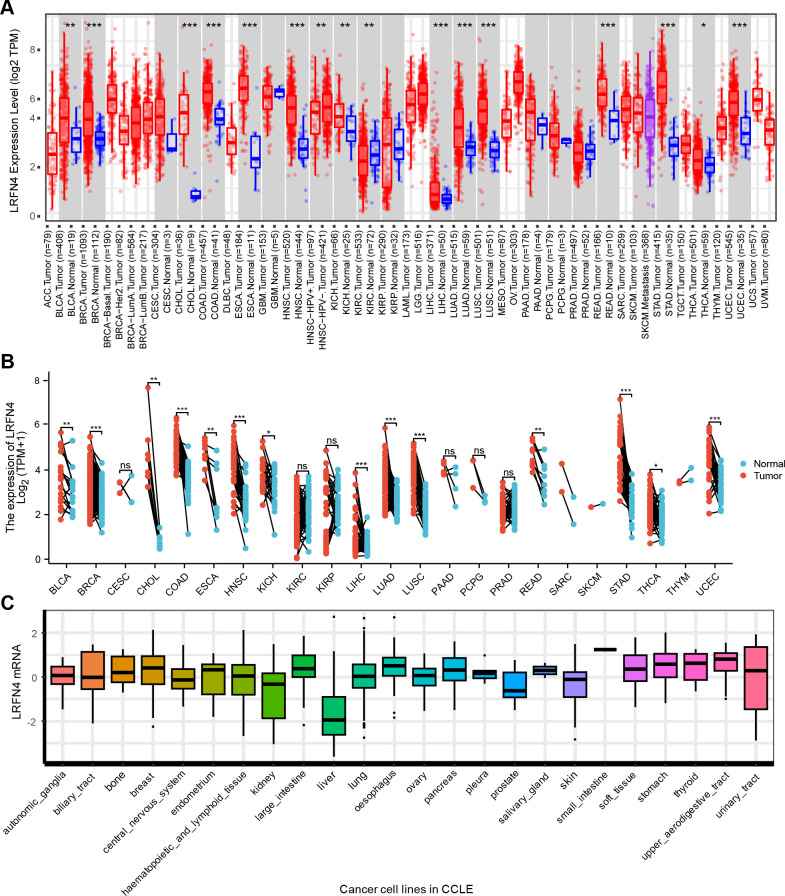
Differential expression of LRFN4. **(A)** Expression levels of LRFN4 (log_2_ TPM) in various tumor and normal tissues across TCGA and GTEx datasets. (Red and blue boxes represent tumor and normal tissues, respectively. The statistical significance of differences between groups is indicated by *P* < 0.05, *P* < 0.01, and *P* < 0.001.) **(B)** Paired analysis of LRFN4 expression in individual tumor types. (Tumor and normal samples are represented by red and blue, respectively. Statistical significance is indicated as described above *P* < 0.05, *P < 0.01, **P < 0.001, ***P < 0.001, and “ns” for non-significant differences.) **(C)** Cancer Cell Line in LRFN4 expression.

This study investigated the potential role of human LRFN4 in tumor development and progression by referencing its mRNA (NM_001363524.2) and protein (NP_001350453.1) sequences. The expression profiles of LRFN4 across multiple cancer types were analyzed using data from The Cancer Genome Atlas (TCGA) (33). As depicted in **Figure 2**, LRFN4 expression was significantly higher in specific cancers including bladder cancer (BLCA), breast cancer (BRCA), cholangiocarcinoma (CHOL), colorectal adenocarcinoma (COAD), esophageal carcinoma (ESCA), head and neck squamous cell carcinoma (HNSC), liver hepatocellular carcinoma (LIHC), lung adenocarcinoma (LUAD), rectum adenocarcinoma (READ), lung squamous cell carcinoma (LUSC), stomach adenocarcinoma (STAD), and uterine corpus endometrial carcinoma (UCEC) compared to matched normal tissues. LRFN4 is also widely expressed in various tumor cell lines. (**Figure 2**).

**Figure 3:**
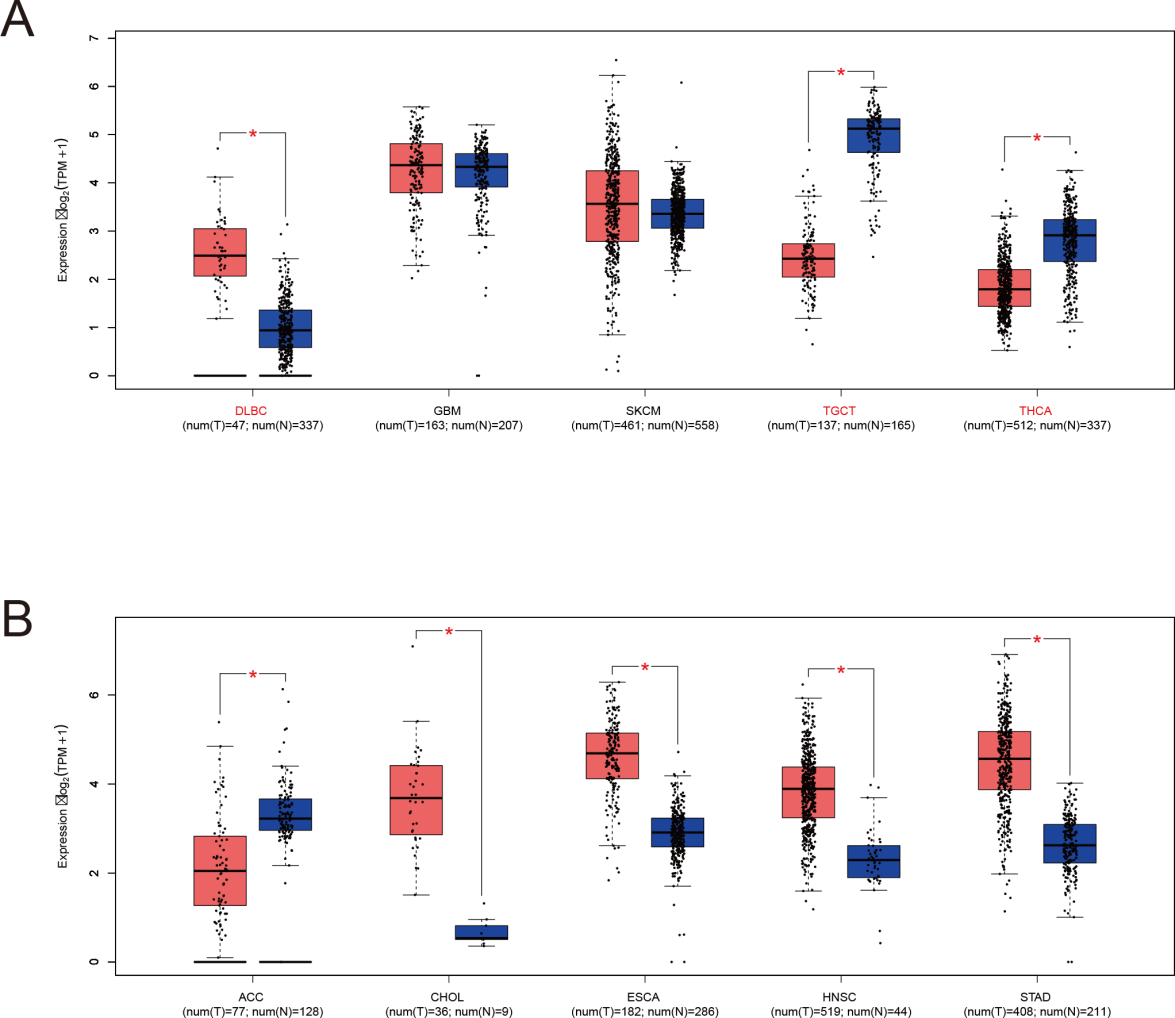
LRFN4 expression in specific tumor and normal tissues based on TCGA and GTEx datasets. **(A)** LRFN4 expression levels (log_2_ TPM + 1) in tumors with sufficient matched normal samples. Red and blue boxes represent tumor and normal tissues, respectively. Sample sizes are indicated below each tissue type (num(T) for tumor and num(N) for normal). **(B)** Analysis of tumor types with limited normal sample availability. Statistical significance is indicated as *P* < 0.05 (*). Bars represent interquartile ranges.

To address the lack of matched normal tissues in certain cancers, normal tissue data from the GTEx database was integrated using the GEPIA platform (http://gepia.cancer-pku.cn). This analysis revealed significant differences in LRFN4 expression for diffuse large B-cell lymphoma (DLBC), glioblastoma (GBM), lower-grade glioma (LGG), skin cutaneous melanoma (SKCM), testicular germ cell tumors (TGCT), and thymoma (THYM) (**Figure 3**). Notably, LRFN4 expression was markedly reduced in TGCT, thyroid carcinoma (THCA), and adrenocortical carcinoma (ACC) tumor tissues.

**Figure 4:**
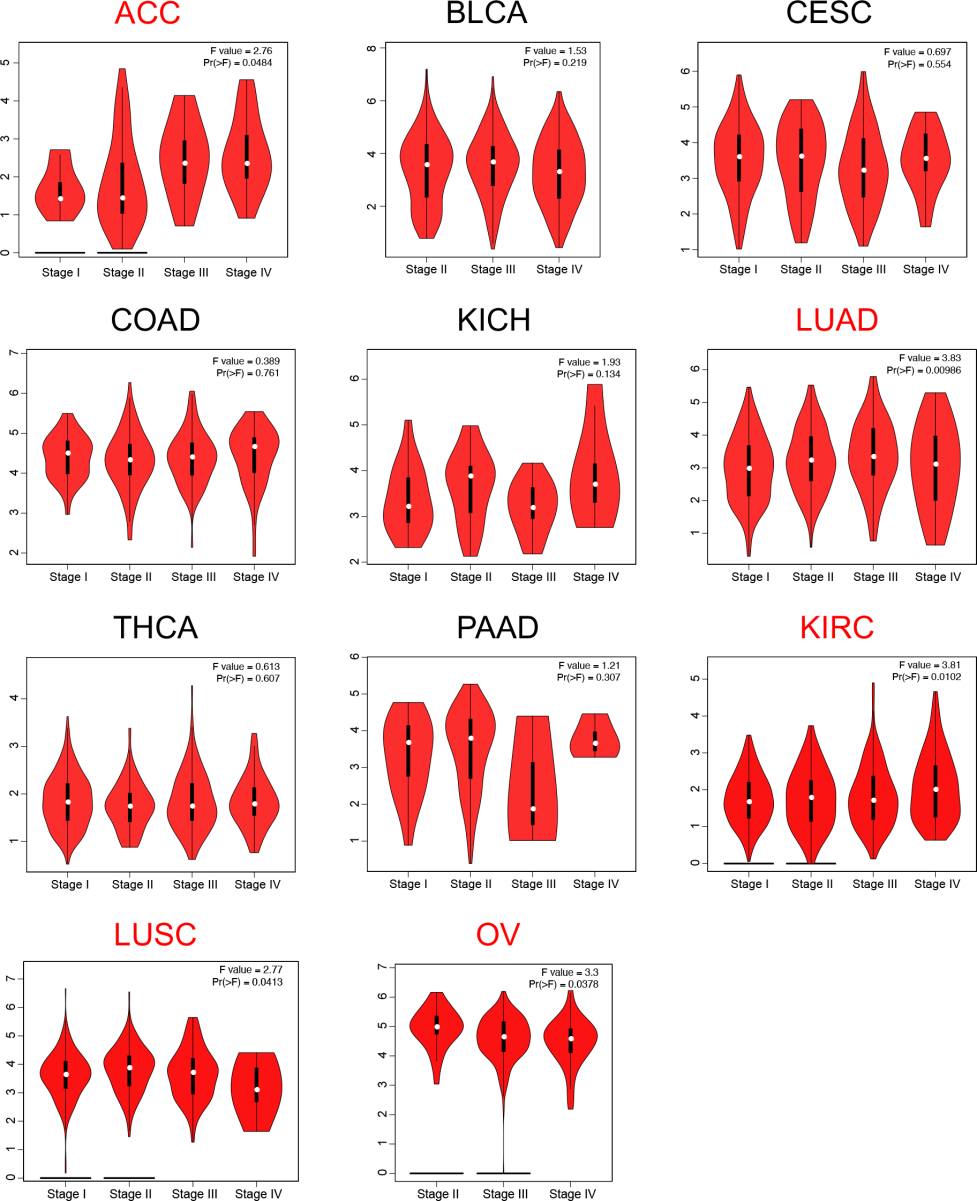
Correlation of LRFN4 expression with pathological staging.

Given the observed pan-cancer overexpression of LRFN4, its association with cancer progression was further elucidated by assessing its correlation with pathological staging. Using the “pathological staging chart” module of GEPIA2, significant correlations between LRFN4 expression and pathological stages were identified in adrenocortical carcinoma (ACC), lung adenocarcinoma (LUAD), kidney renal clear cell carcinoma (KIRC), lung squamous cell carcinoma (LUSC), and ovarian cancer (OV) (**Figure 4**). No significant correlations were observed in other cancer types. These findings highlight the potential value of LRFN4 as a biomarker for cancer progression and its implications for therapeutic strategy formulation.

### 3.2 Diagnosis value of LRFN4 across cancers

**Figure 5:**
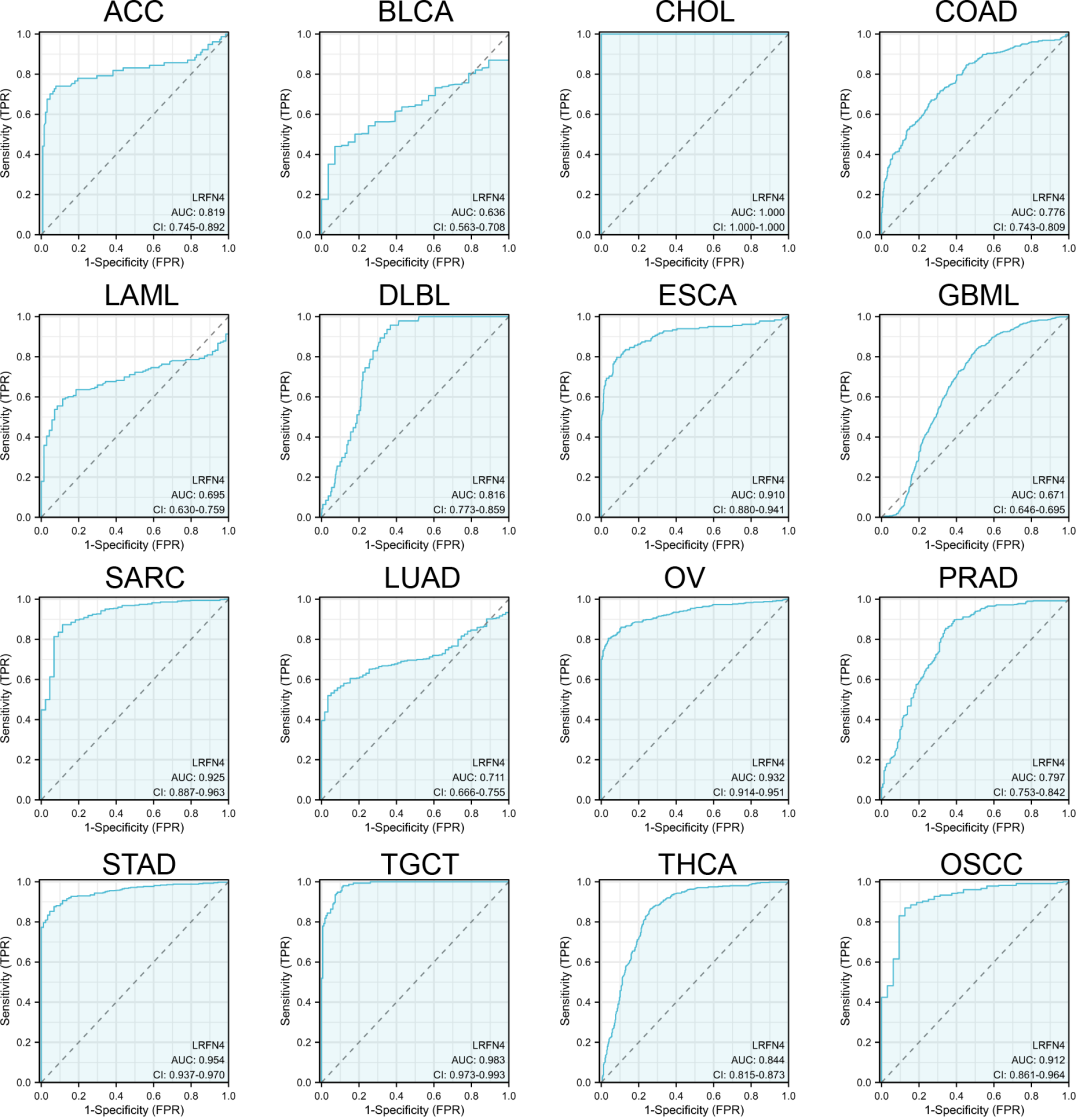
Diagnostic potential of LRFN4 assessed by ROC analysis. Receiver Operating Characteristic (ROC) curves assessing the diagnostic performance of LRFN4 in distinguishing tumors from normal tissues. The Area Under the Curve (AUC) values are reported for various cancers, with values closer to 1 indicating better diagnostic accuracy.

Receiver Operating Characteristic (ROC) curves (**Figure 5**) were constructed to assess LRFN4’s diagnostic potential in distinguishing tumors from normal tissues. LRFN4 demonstrated promising diagnostic utility across multiple cancers, with Area Under the Curve (AUC) values: ACC (0.819), BLCA (0.636), CHOL (1.000), COAD (0.776), LAML (0.695), DLBCL (0.816), ESCA (0.910), GBML (0.671), SARC (0.925), LUAD (0.711), OV (0.932), PRAD (0.797), STAD (0.954), TGCT (0.983), THCA (0.844), and OSCC (0.912), with AUC values closer to 1 indicating superior predictive performance.

### 3.3 Prognostic significance of LRFN4 across cancers

**Figure 6:**
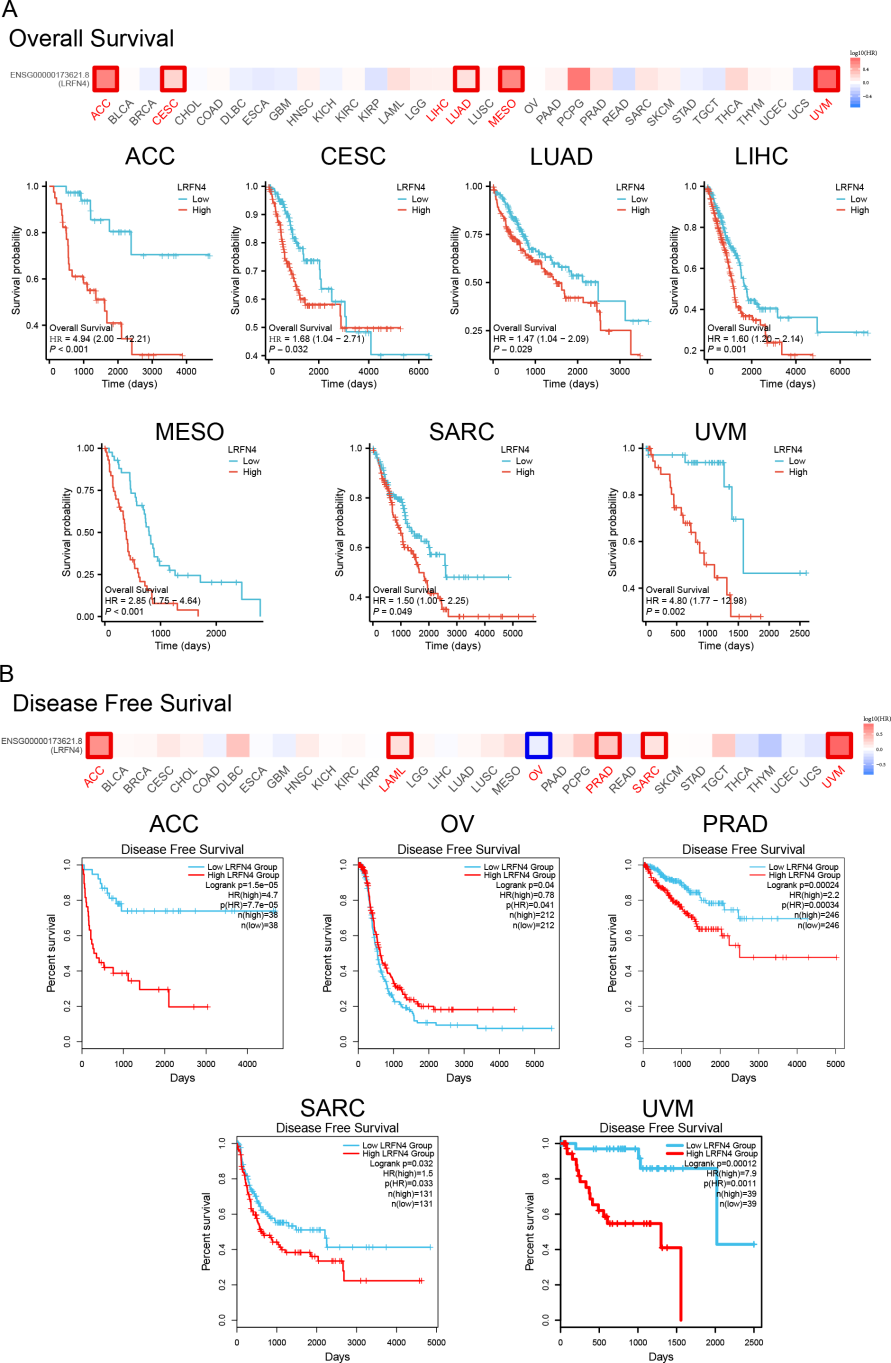
Prognostic significance of LRFN4 expression in cancer. **(A)** Kaplan-Meier survival analysis showing overall survival (OS) stratified by high and low LRFN4 expression in various cancers. **(B)** Disease-free survival (DFS) analysis depicting the relationship between LRFN4 expression and DFS outcomes.

Tumor samples were stratified into high- and low-expression groups for analyses of overall survival (OS) and disease-free survival (DFS) using TCGA datasets. Results demonstrated significant associations of high LRFN4 expression with poor OS in ACC (*P* < 0.001), CESC (*P* = 0.032), LUAD (*P* = 0.029), and LIHC (*P* = 0.001) (**Figure 6A**). DFS analysis (**Figure 6B**) showed correlation with worse outcomes for high LRFN4 expression in ACC (*P* = 1.5e-05), PRAD (*P* = 0.00024), SARC (*P* = 0.0032), and UVM (*P* = 0.00012), while low LRFN4 expression was associated with poor DFS in OV (*P* = 0.004) (34).

**Figure 7:**
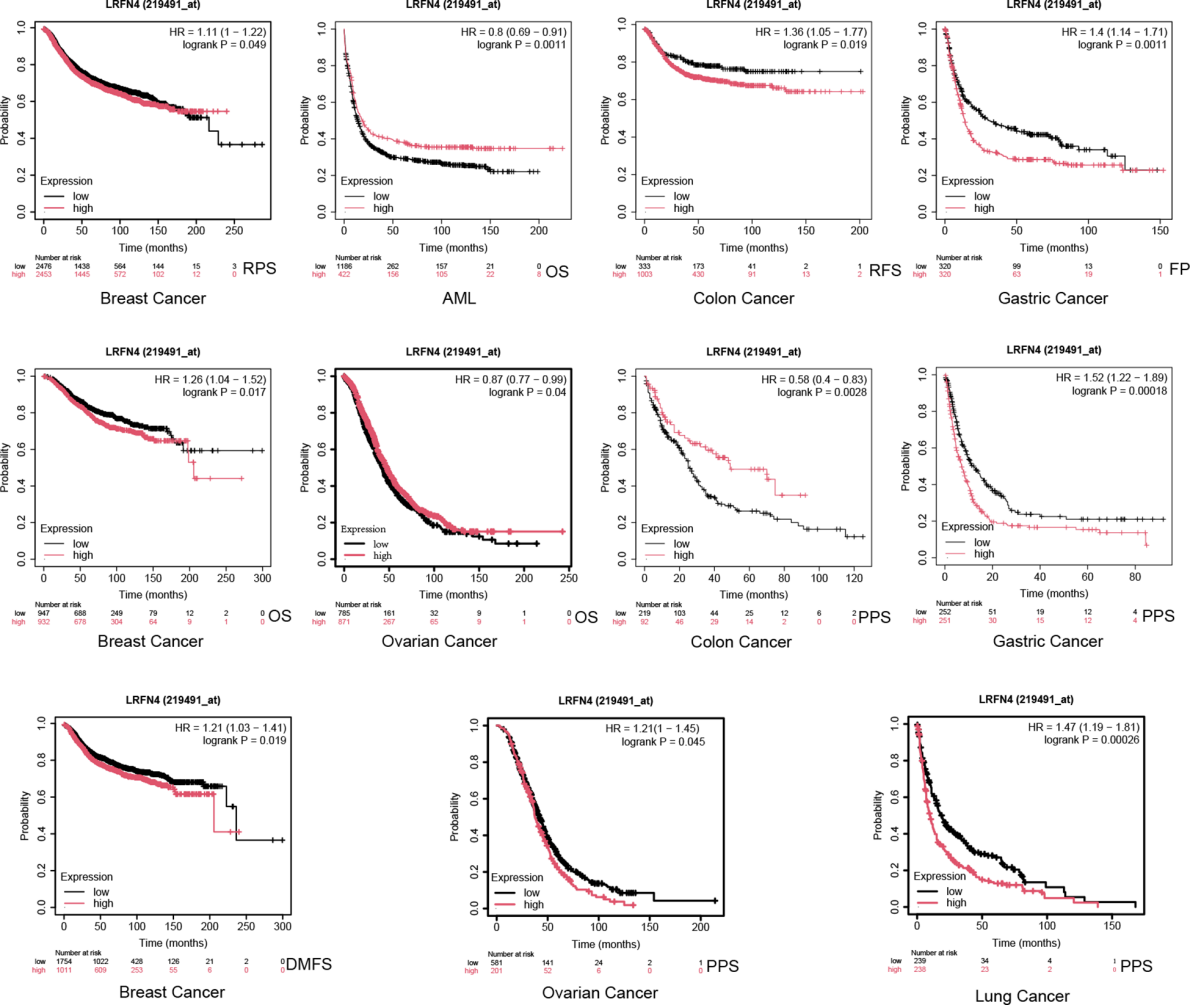
Additional survival analyses of LRFN4 expression in cancer. Kaplan-Meier plots derived from the GEO database were utilized to evaluate the prognostic significance of LRFN4 expression in relation to overall survival (OS), distant metastasis-free survival (DMFS), and recurrence-free survival (RFS) across a spectrum of cancer types.

Using the Kaplan-Meier plotter tool, high LRFN4 expression was associated with poor OS (*P* = 0.017), distant metastasis-free survival (DMFS, *P* = 0.019), and recurrence-free survival (RFS, *P* = 0.049) in breast cancer, and with progression-free interval (PFI) in various other cancer types. Conversely, low LRFN4 expression was associated with poor OS in AML and OV (*P* < 0.05). High LRFN4 expression correlated with poor prognosis in gastric cancer (FP, *P* = 0.0011; PPS, *P* = 0.00018), ovarian cancer (RFS, *P* = 0.045), colon cancer (relapse-free survival, *P* = 0.0019), and lung cancer (*P* = 0.00026) (**Figure 7**). Notably, low LRFN4 expression was related to poor prognosis in colon cancer (*P* = 0.0028), but showed no significant correlation with OS, PFS, RFS, or DSS in liver cancer. These findings suggest that LRFN4’s prognostic significance is tumor-type specific, indicating its potential as a prognostic biomarker across various malignancies.

### 3.4 Associations of LRFN4 with immune and molecular subtypes

**Figure 8:**
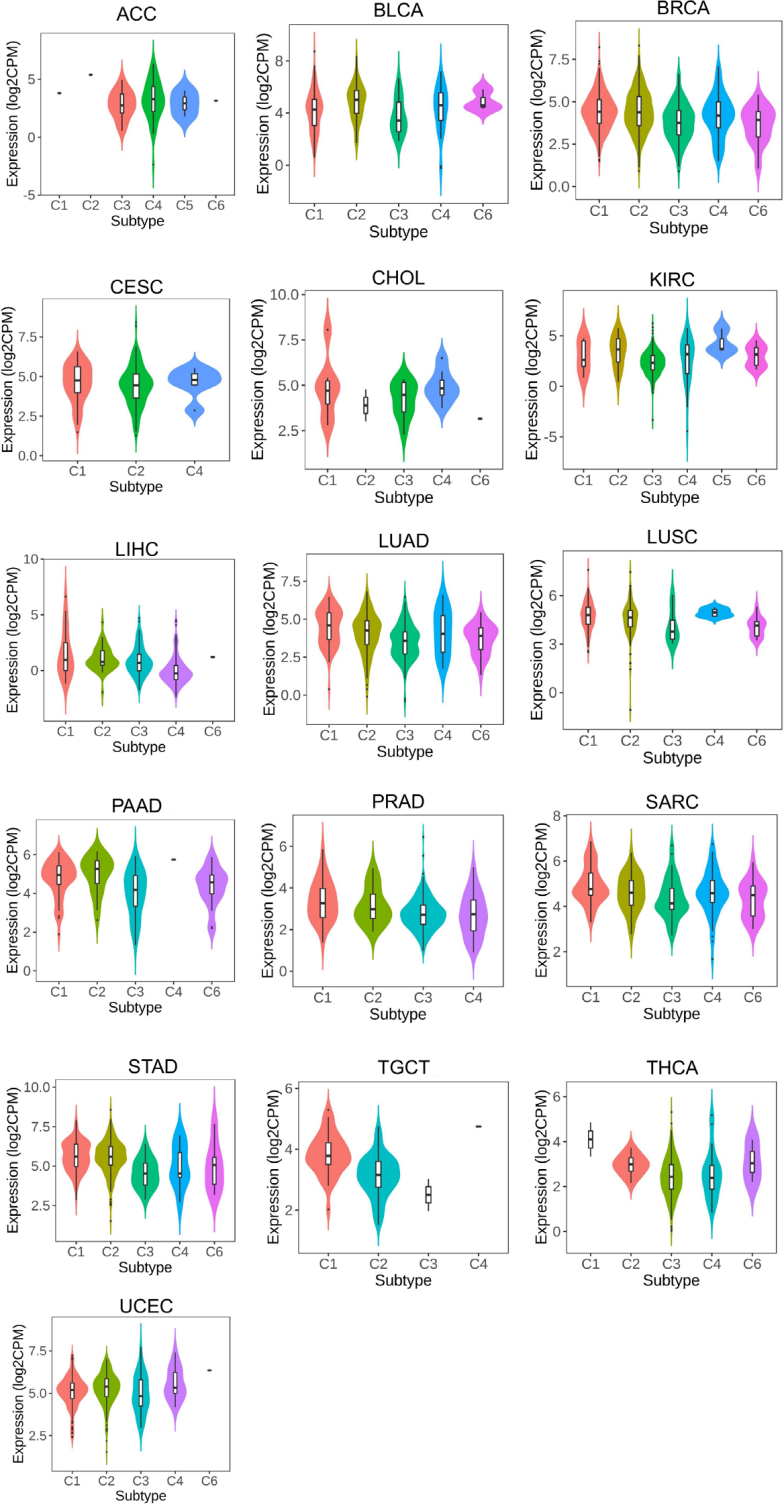
LRFN4 expression and immune subtypes correlation. C1, wound healing.C2, IFN-gamma dominant. C3, inflammatory. C4, lymphocyte depleted. C5, immunologically quiet.C6, TGF-β dominant.

Using the TISIDB portal, the association of LRFN4 with immune subtypes was examined across various cancers, including ACC, BLCA, BRCA, CESC, CHOL, KIRP, KIRC, LIHC, LUAD, LUSC, PAAD, PRAD, SARC, STAD, TGCT, THCA, and UCEC (**Figure 8**). Significantly, LRFN4 expression was associated with immune subtypes, indicating its potential role in regulating immune responses in cancers.

**Figure 9:**
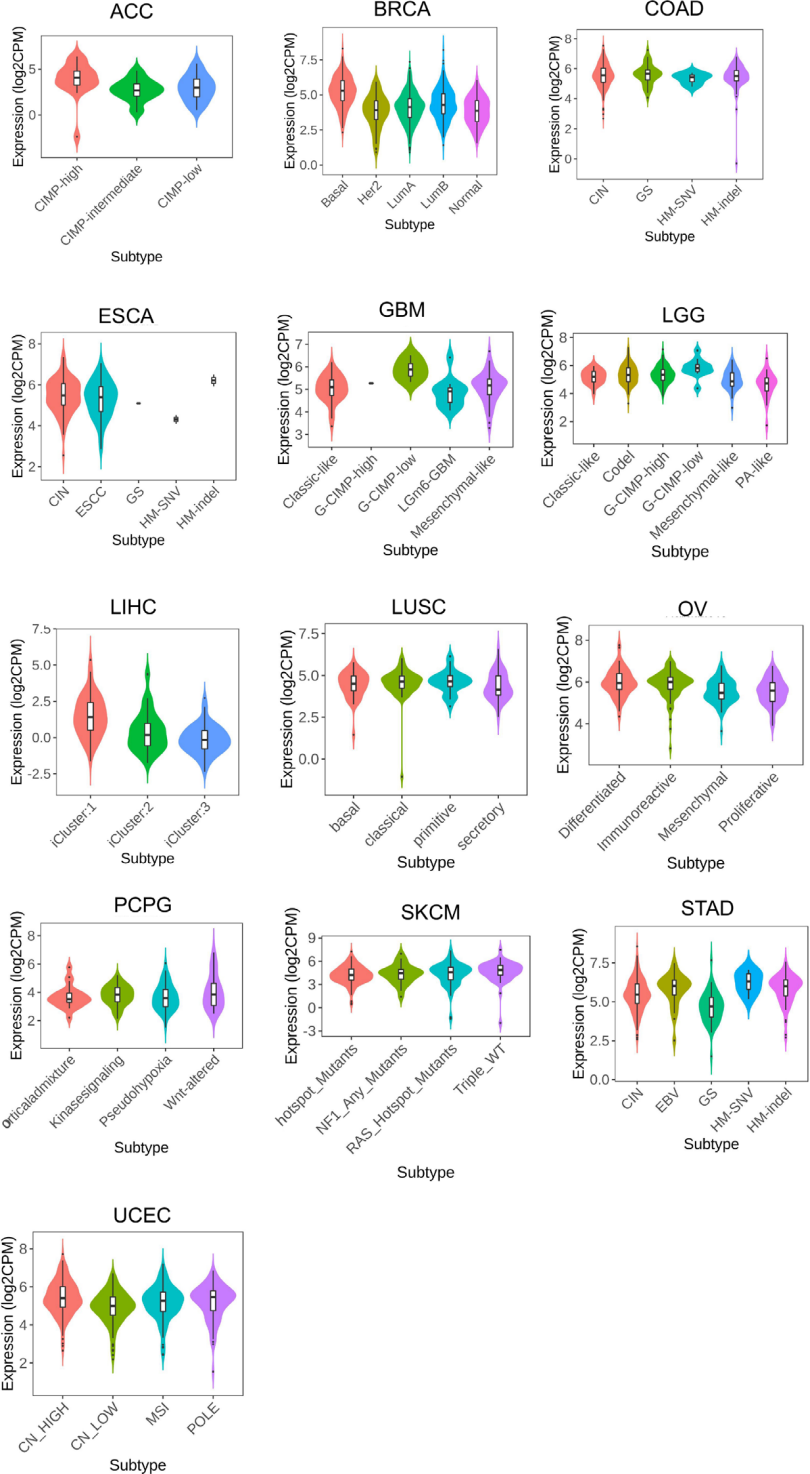
LRFN4 expression and molecular subtypes correlation.

LRFN4 expression was found to differentiate among different molecular types in cancer types such as ACC, BRCA, COAD, ESCA, GBM, LGG, LIHC, LUSC, OV, PCPG, SKCM, STAD, and UCEC (**Figure 9**). These findings suggest a close relationship between LRFN4 expression and molecular subtypes, contributing to the molecular classification of tumors.Overall, these results imply the potential of LRFN4 as a biomarker for immune and molecular subtype classification of various cancers.

### 3.5 Relationships of LRFN4 with TMB, MSI, and ESTIMATE scores

**Figure 10:**
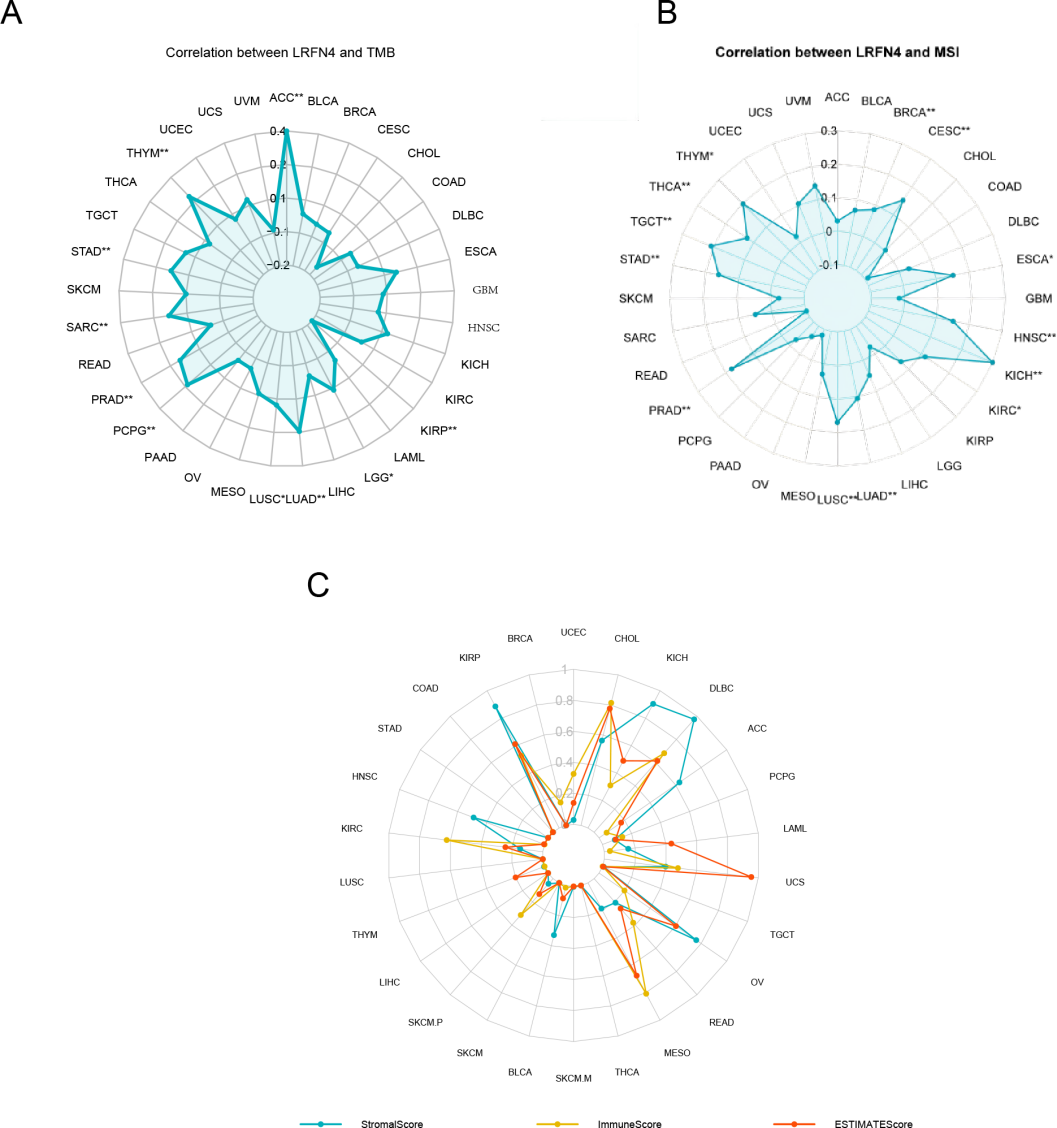
LRFN4 expression and biomarkers of therapeutic efficacy. Correlation analysis between LRFN4 expression and key therapeutic biomarkers, including **(A)** Tumor Mutational Burden (TMB), **(B)** Microsatellite Instability (MSI), **(C)** the ESTIMATE score.

The expression of LRFN4 was examined for its relationship with TMB, which affects immunotherapy efficacy. LRFN4 expression correlated positively with TMB in ACC, LGG, LUAD, LUSC, PCPG, PRAD, SARC, STAD, and THYM, and negatively with KIRP (**Figure 10A**).A positive correlation was found between MSI and LRFN4 expression in BRCA, CESC, ESCA, HNSC, KICH, LUAD, LUSC, PRAD, STAD, TGCT, THCA, and THYM (**Figure 10B**). The relationship between LRFN4 and the three scores from the ESTIMATE algorithm was examined. A significant positive correlation was confirmed in UCEC, CHOL, KICH, DLBC, ACC, PCPG, LAML, UCS, OV, READ, MESO, BLCA, SKCM.P, KIRC, HNSC, and KIRP (**Figure 10C**), suggesting LRFN4’s potential role in anti-tumor immunity through influencing the TME composition.

### 3.6 Data analysis of LRFN4 genetic alterations

**Figure 11:**
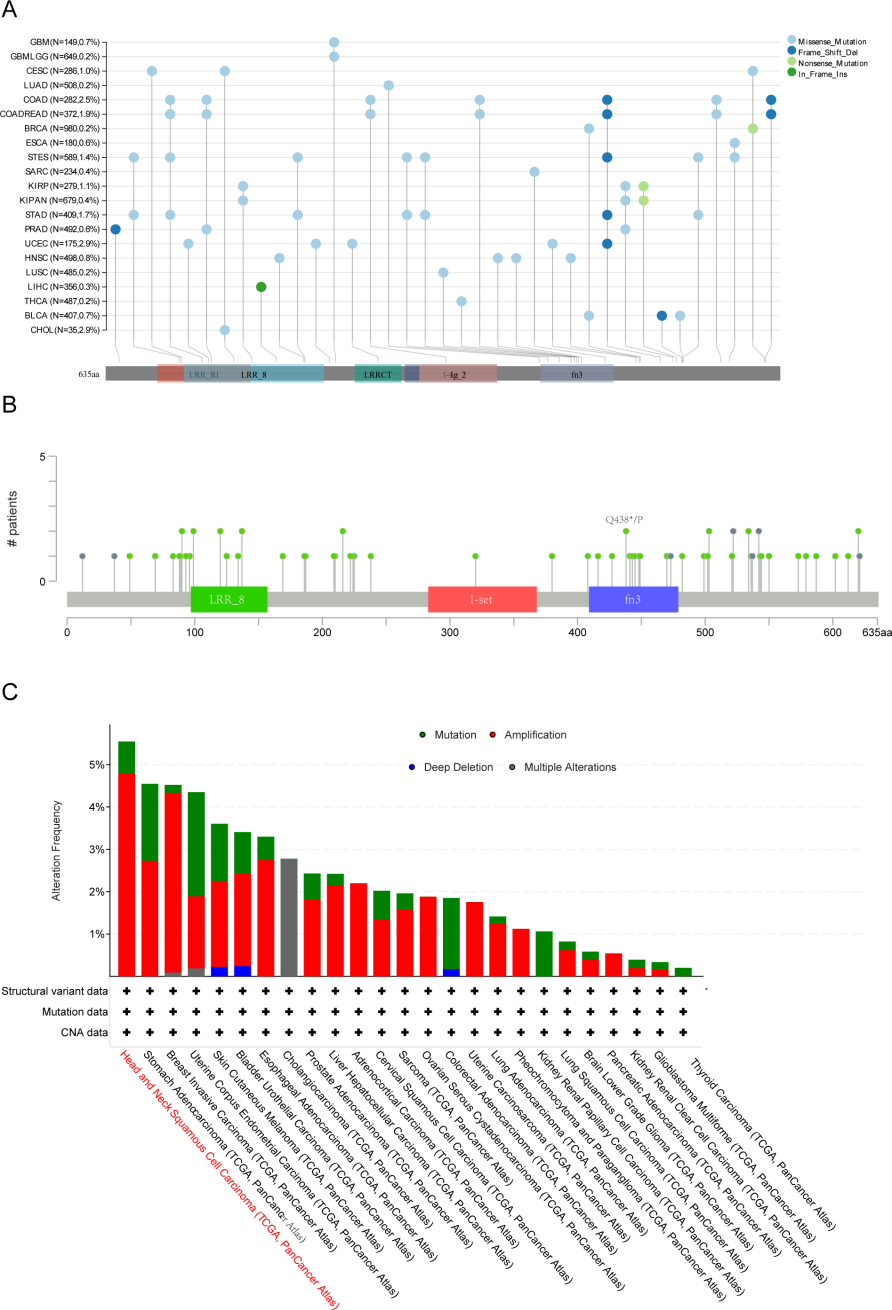
Genetic alteration analysis of LRFN4 across pan-cancer cohort. **(A)** Mutation hotspot mapping in the LRFN4 gene, with mutation sites mapped to functional protein domains. **(B)** 3D structural model of LRFN4 showing mutation locations. **(C)** Mutation frequency of LRFN4 across cancer types in the TCGA cohort.

Using TCGA cohort data, we analyzed the genetic alteration status of LRFN4 across multiple tumor types. As depicted in **Figure 11C**, the highest frequency of LRFN4 mutations, surpassing 5%, occurred in Head and Neck Squamous Cell Carcinoma (HNSCC), where alterations were predominantly classified as “mutation.” In gastric cancer, “amplification” represented the main type of copy number alteration (CNA), with mutation frequencies approximating 2% (**Figure 11C**) (35). These results indicate a widespread spectrum of genetic changes of LRFN4 across different cancers, suggesting its potential role in tumorigenesis. A comprehensive analysis of LRFN4 gene alteration types, locations, and cases is shown in **Figure 11A**. Amplification was observed as the most prevalent alteration type. Domain-specific changes, such as Q48*p of the fn3 domain (**Figure 11B**), were detected in isolated cases of lung adenocarcinoma (LUAD) and stomach adenocarcinoma (STAD), potentially leading to missense mutations in the LRFN4 gene, highlighting the diverse genetic landscapes associated with LRFN4 alterations.

To explore the clinical relevance, we examined the association between LRFN4 alterations and survival outcomes across various cancers. Despite the unavailability of certain data, such as rectum adenocarcinoma (READ) on the cBioPortal platform (36), the prevalence of LRFN4 alterations in digestive system malignancies was evaluated, showing an overall incidence of 3% (**Supplementary Figure S1A**).

The impact of LRFN4 gene alterations on expression levels was also evaluated. (**Supplementary Figure S1B**) For instance, copy number variations (CNVs) (37) were less frequent in colorectal adenocarcinoma (COAD) and pancreatic adenocarcinoma (PAAD). Notably, the correlation between LRFN4 expression levels and clinical features in COAD and PAAD was found to be weaker, as these tumor types exhibited fewer CNVs. These findings suggest that genomic alterations of LRFN4 are common across cancers and may affect its expression, contributing to the regulation of cancer progression.

### 3.7 Pan-cancer correlations between LRFN4 and immune genes

**Figure 12:**
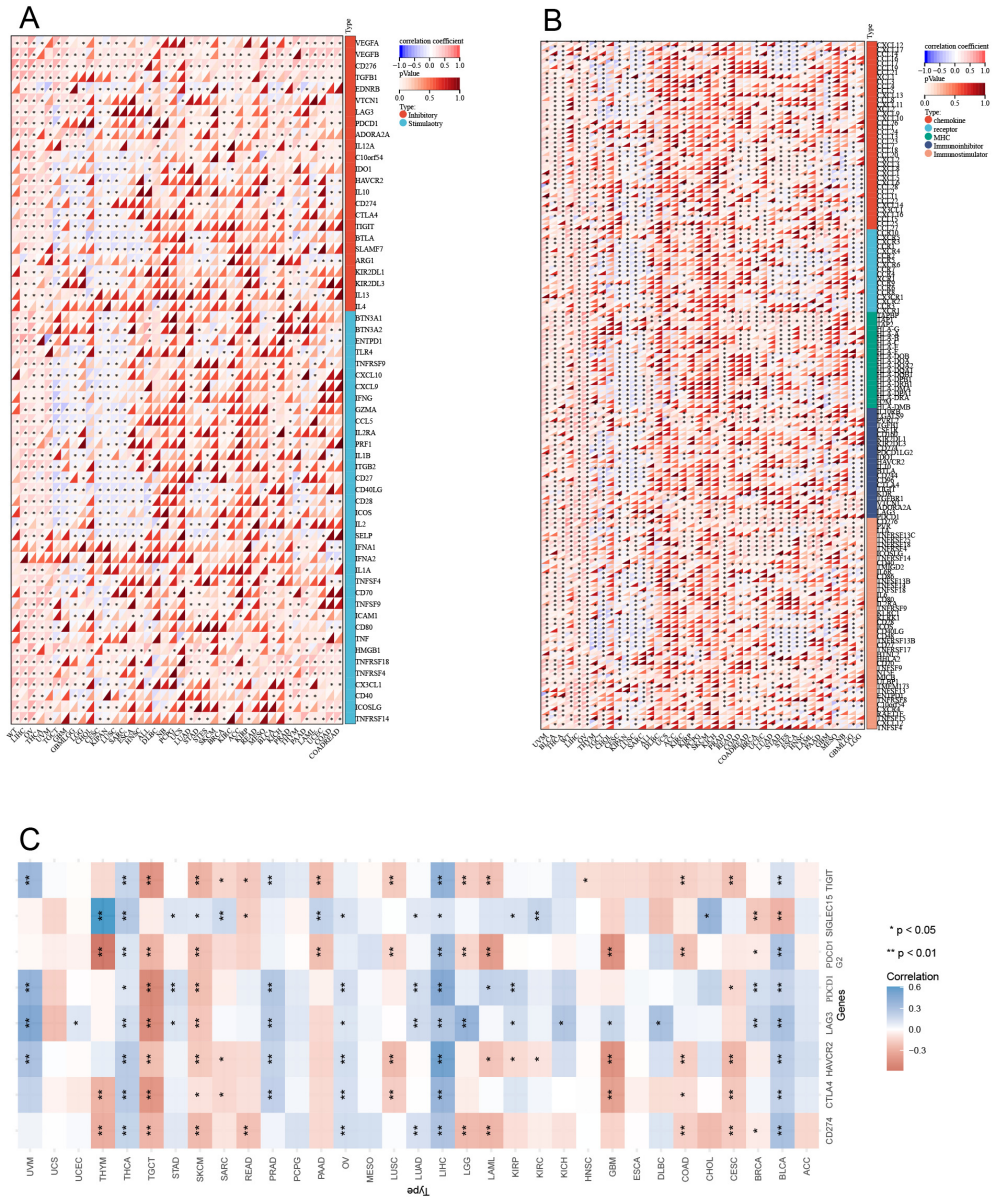
Correlation between LRFN4 and immune-related genes. **(A)** Pearson correlation analysis of LRFN4 with inhibitory and stimulatory immune checkpoint genes. **(B)** LRFN4 correlation with immune pathway marker gene sets. **(C)** Expression of LRFN4 in relation to immune checkpoint-associated genes across cancer types.

Co-expression analyses of LRFN4 and ICP genes were performed across 33 tumor types. As shown in **Figure 12A**, almost all ICP-related genes exhibited significant co-expressed with LRFN4, except in LUSC and SARC, where a negative correlation was observed. In other tumor types, a positive correlation with LRFN4 was found (*P* < 0.05). These findings imply that high LRFN4 expression could predict immunotherapy responses targeting ICP genes, indicating its potential as a novel therapeutic target in immunotherapy (5). Interestingly, LRFN4 showed limited co-expression with ICP genes in READ and MESO, suggesting suboptimal immunotherapy responses for patients with high LRFN4 expression in these tumors. These results highlight LRFN4’s complex role in the tumor immune microenvironment and its potential as a prognostic biomarker or therapeutic target in human cancer immunotherapy.

To further explore LRFN4’s role in immune regulation, Pearson correlation coefficients between ENSG00000173621 (LRFN4) and marker genes of five major immune pathways were calculated (38). Except for CESC, KIPAN, and LUSC where negative correlations were observed, most immune-related genes positively correlated with LRFN4 expression across tumor types (*P* < 0.05) (**Figure 12B**). These findings suggest an intricate link between LRFN4 and immune microenvironment, highlighting its cancer-specific influence on immune pathway activation. A comprehensive and extensive correlation has been identified between LRFN4 and immune checkpoint genes. (**Figure 12C**).

### 3.8 Correlation between LRFN4 and immune cell infiltration in the tumor microenvironment

**Figure 13:**
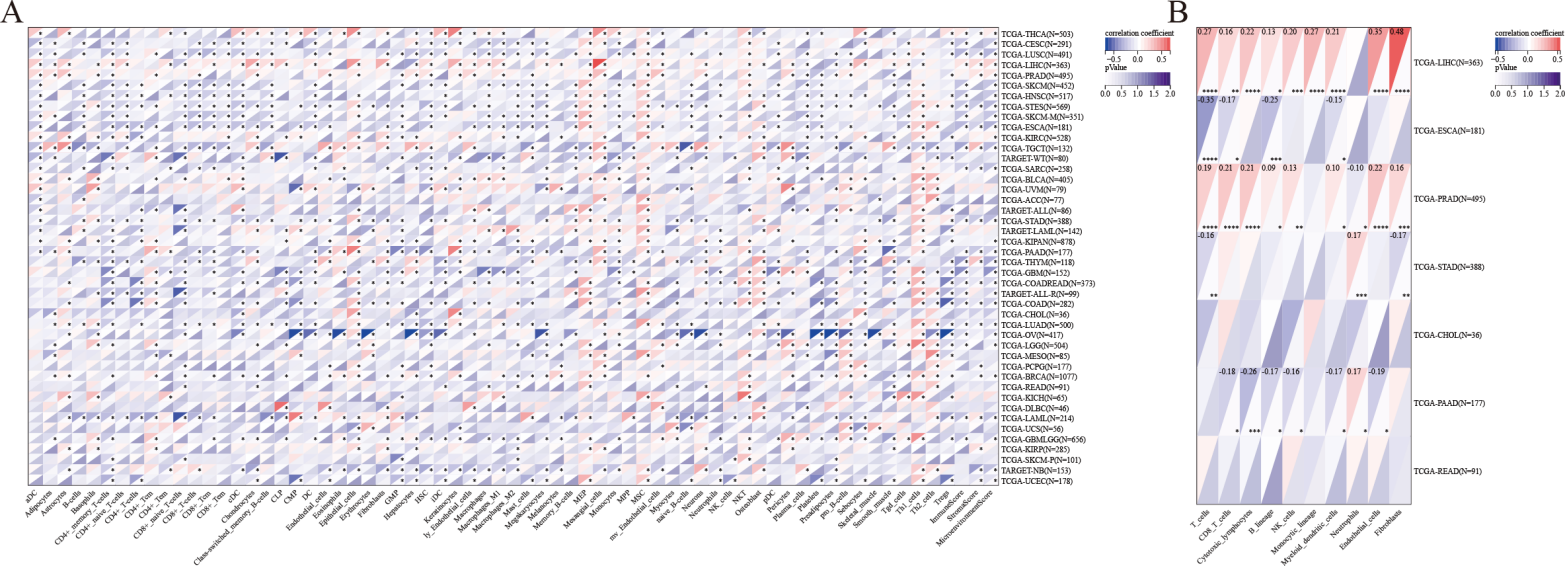
Immune infiltration analysis. **(A)** Immune infiltration scores for 67 immune and stromal cell types calculated using the xCell method. **(B)** Correlation between LRFN4 expression and immune cell populations specifically analyzed in seven digestive system cancers. *P < 0.05, **P < 0.01, ***P < 0.001, ****p < 0.0001.

Significant correlations were identified between LRFN4 expression and immune cell infiltration across all 44 cancer types (39, 40), showing notable positive associations with certain immune cell types (e.g., MEP cells, MSC cells, Th1 cells, Th2 cells) and significant negative correlations with others (*P* < 0.05) (**Figure 13A**). The deconvo_mcpcounter method (27) was employed to further analyze the correlation between LRFN4 expression and immune cell populations in seven digestive system cancers:

Hepatocellular carcinoma (LIHC): Significant positive correlations were observed between LRFN4 and various immune cell types (T cells, CD8+ T cells, cytotoxic lymphocytes, B lineage cells, NK cells, monocytic lineage cells, myeloid dendritic cells, endothelial cells, and fibroblasts).Esophageal cancer (ESCA): Correlations were found with T cells, CD8+ T cells, B lineage cells, and myeloid dendritic cells. Prostate adenocarcinoma (PRAD): LRFN4 was associated with T cells, CD8+ T cells, cytotoxic lymphocytes, B lineage cells, NK cells, myeloid dendritic cells, neutrophils, endothelial cells, and fibroblasts. Stomach adenocarcinoma (STAD): Significant associations were observed with T cells, neutrophils, and fibroblasts. Pancreatic adenocarcinoma (PAAD): Positive correlations were noted with CD8+ T cells, cytotoxic lymphocytes, B lineage cells, NK cells, myeloid dendritic cells, neutrophils, endothelial cells, and fibroblasts (**Figure 13B**).

### 3.9 Enrichment analysis of LRFN4-associated correlations

To elucidate the molecular mechanisms of LRFN4 in tumorigenesis, analyses for LRFN4-binding proteins and correlated genes were performed, followed by pathway enrichment studies.

**Figure 14:**
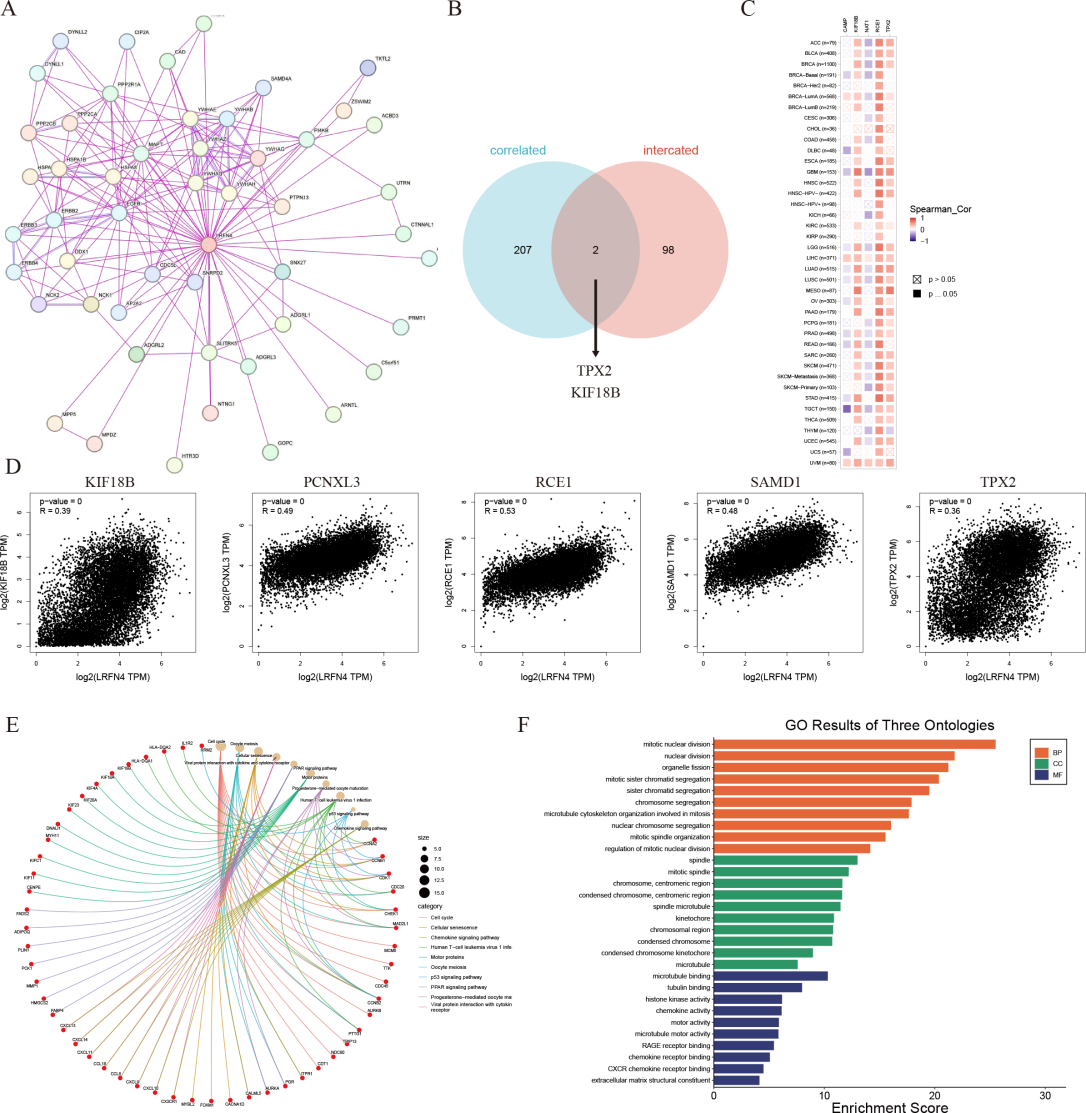
LRFN4-related gene enrichment analysis. **(A)** Protein-protein interaction (PPI) network for LRFN4 using STRING. **(B)** Venn diagram showing overlap between LRFN4-binding proteins and LRFN4-correlated genes. **(C)** Heatmap of LRFN4-correlated genes using TIMER. **(D)** Scatter plot of Pearson correlation analysis between LRFN4 and top 100 correlated genes from GEPIA2. **(E)** Kyoto Encyclopedia of Genes and Genomes (KEGG) pathway enrichment analysis of LRFN4-related genes. **(F)** Gene Ontology (GO) enrichment analysis of LRFN4-related genes.

Using STRING, 50 LRFN4-binding proteins (supported by experimental evidence) were identified, with their interaction network shown in **Figure 14A** (41). Venn diagram analysis revealed TPX2 and KIF18B as common genes between LRFN4-binding proteins and correlated gene sets. (**Figure 14B**) The heatmap (**Figure 14C**) indicated positive correlations of LRFN4 with these five genes across most analyzed cancer types. From TCGA tumor expression data via GEPIA2, the top 100 genes positively correlated with LRFN4 expression were determined, among which TPX2 (R = 0.36), KIF18B (R = 0.39), RCE1 (R = 0.53), PCNXL3 (R = 0.49), and SAMD1 (R = 0.48) showed significant correlations (*P* < 0.001) (**Figure 14D**). KEGG and GO enrichment analyses were further performed by integrating the LRFN4-binding protein dataset with the top 100 LRFN4-correlated genes. KEGG enrichment (**Figure 14E**, (42) suggested LRFN4’s potential involvement in tumorigenesis through pathways like the cell cycle, cell senescence, and chemokine signaling. GO analysis (**Figure 14F**) showed that associated genes participate in cell proliferation processes, including mitosis and chemokine signaling, underscoring LRFN4’s essential role in cell mitosis and proliferation. Overall, these results highlight LRFN4’s potential as a key regulator in tumor pathogenesis and its value in cancer prognosis and therapy.

### 3.10 Impact of LRFN4 protein on clinicopathologic features and immune infiltration in STAD

**Figure 15:**
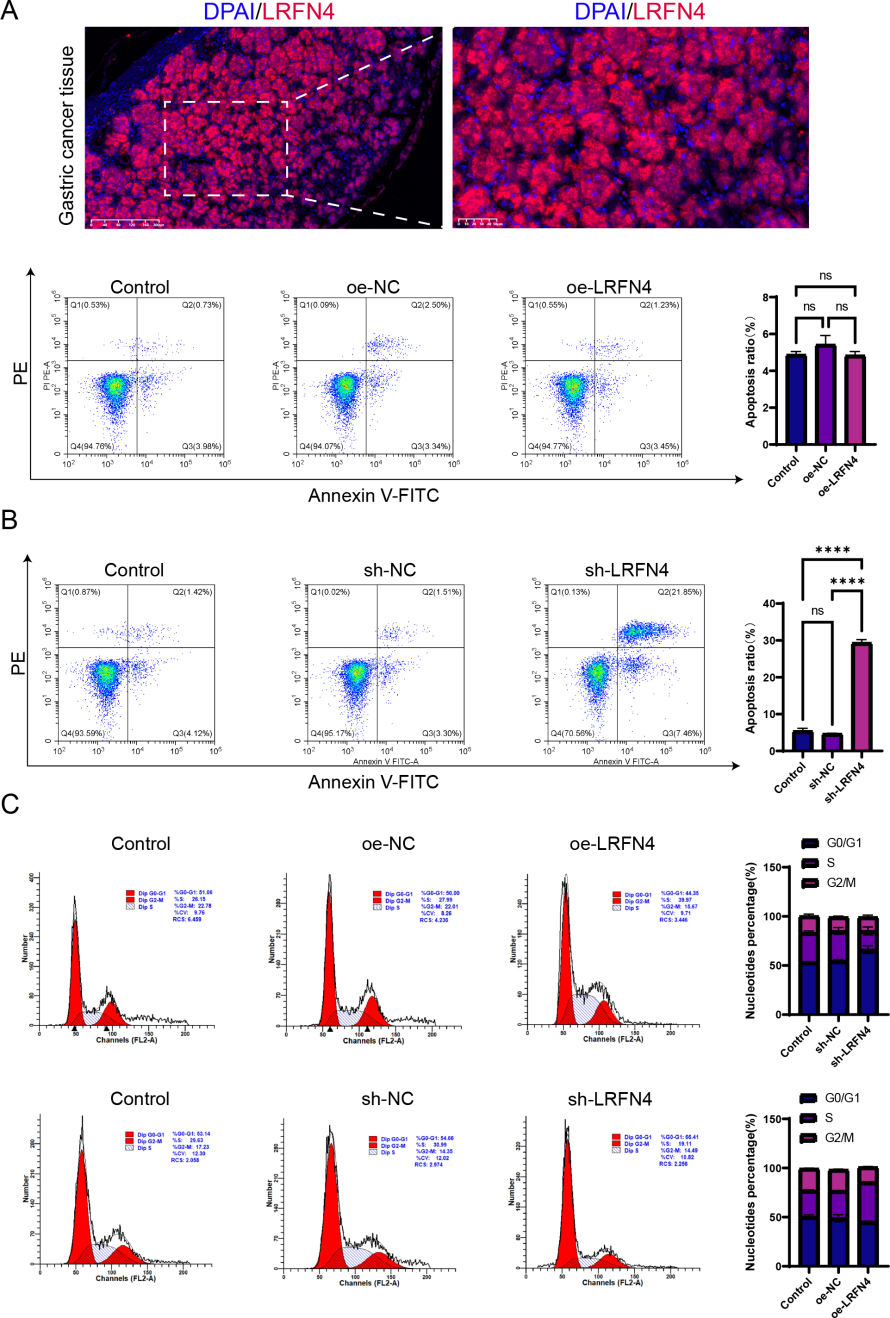
Influence of LRFN4 on apoptosis and cell cycle dynamics in STAD cells. Fluorescence-based multiplex immunohistochemistry (mIHC) analysis of LRFN4 protein expression in stomach adenocarcinoma tissues, correlating with clinicopathological features and immune cell infiltration. **(A)** Flow cytometry analysis demonstrating the effects of LRFN4 knockdown and overexpression on apoptosis and cell cycle progression in stomach adenocarcinoma (STAD) cells **(B, C)**. NS, Not Significant. ****p < 0.0001.

We detected the expression of LRFN4 protein in 80 cases of STAD tissues using fluorescence-based multiplex immunohistochemistry (mIHC) and confirmed these findings. LRFN4 protein was localized in both the cytoplasm and the interstitium (**Figure 15A**). (**Table 1**) Furthermore, LRFN4 protein expression exhibited significant associations with vascular invasion, tumor size, and TNM stage (**Table 2**).

**Table 1:** Comparison of baseline data between the patient group and the healthy control group.

	Cancer Group (n = 80)	Para - cancer Group (n = 80)	Statistical Value	P Value
Number of LRFN4 - positive cells	80.5 (41.5,136.5)	108 (76,140.25)	Z=1.818	0.003^*^
Total number of cells	13128.5 (5844,18149.5)	10157.5 (7386.75,13621.75)	Z=1.660	0.008^*^
Positive rate of LRFN4	0.78% (0.34%,1.35%)	0.94% (0.65%,1.54%)	Z=1.581	0.013^*^

*P < 0.05.

**Table 2:** Comparison of LRFN4 positive rates.

			LRFN4 Positive Rates			
		n	Low	High	X^2^ Statistical Value	P
Total		80	41 (51.2)	39 (48.8)	–	–
Gender	Male	53	29 (54.7)	24 (45.3)	0.756	0.385
	Female	27	12 (44.4)	15 (55.6)		
Age	<55	37	15 (40.5)	22 (59.5)	3.160	0.075^*^
	≥55	43	26 (60.5)	17 (39.5)		
Immunohistochemistry	None	41	26 (63.4)	15 (36.6)	4.981	0.026^*^
	Yes	39	15 (38.5)	24 (61.5)		
TNM Stage	T1+T2	23	17 (73.9)	6 (26.1)	6.636	0.010^*^
	T3+T4	57	24 (42.1)	33 (57.9)		
Stage	I+II	15	12 (80)	3 (20)	6.108	0.013^*^
	III+IV	65	29 (44.6)	36 (55.4)		
Survival Status	No	44	29 (65.9)	15 (34.1)	8.410	0.004^*^
	Yes	36	12 (33.3)	24 (66.7)		

*P < 0.05.

### 3.11 Influence of LRFN4 protein on apoptosis and cell cycle kinetics in STAD cells

Flow cytometry was employed to detect specific alterations in apoptosis and cell cycle progression in STAD cells following the knockdown and overexpression of LRFN4. The results indicate that the knockdown of LRFN4 significantly promotes apoptosis in STAD cells and markedly reduces S phase arrest. This suggests that LRFN4 may enhance the survival of gastric cancer cells by inhibiting apoptosis and may play a role in regulating the cell cycle, particularly during the S phase. Its knockdown impedes the normal progression through the S phase, thereby impacting the cells’ proliferative capacity. Future studies should further investigate the molecular mechanisms by which LRFN4 regulates apoptosis and cell cycle regulation. (**Figures 15B, C**).

The Western Blot analysis indicated a positive correlation between high LRFN4 expression and the levels of cyclin D1 and CDK4, suggesting that LRFN4 may contribute to the promotion of cell cycle progression. In contrast, a negative correlation was observed between elevated LRFN4 expression and cleaved-caspase-3 expression, indicating that LRFN4 might inhibit apoptosis by suppressing caspase-3 activation. These findings underscore the potential of LRFN4 as a therapeutic target in cancer treatment (**Figure 1**).

## 4 Discussion

Cancer - related research has consistently remained a primary focus in the contemporary medical field. Data from the TCGA, CCLE platforms, and GEO databases were utilized, along with molecular characteristics such as gene expression, genetic alterations, DNA methylation, and protein phosphorylation, to conduct an in-depth examination of the LRFN4 gene across 33 distinct tumor types. This investigation focused on the relationship between LRFN4 expression and tumor immunity, specifically through infiltration correlation analysis. This study represents the first comprehensive pan-cancer analysis of LRFN4, aiming to systematically characterize its expression pattern and prognostic significance. The results reveal that LRFN4 is significantly differentially expressed across various tumors, with expression levels substantially higher than those in paired normal tissues. These findings confirm the widespread occurrence of aberrant LRFN4 expression in cancers. It is postulated that the LRFN4 protein could serve as a potential biomarker for screening multiple tumors and likely plays a critical role in tumor initiation and progression. As a member of the leucine - rich repeat and fibronectin type III domain-containing family and a type I transmembrane glycoprotein, LRFN4 may interact with diverse cellular components within the tumor microenvironment (TME). For example, its extracellular leucine-rich repeats might interact with growth factors or cell-surface receptors, while the fibronectin type III domain could participate in cell-matrix or cell-cell interactions. These interactions potentially activate oncogenic signaling pathways, facilitating cancer development. Previous reports on lung, colon, and breast cancers have demonstrated that LRFN4’s ability to enhance cell proliferation, invasion, and chemotherapy resistance, suggesting its involvement in multiple oncogenic pathways (43, 44). A structurally similar protein has been shown to promote cell proliferation by binding to the epidermal growth factor receptor and activating the downstream PI3K-AKT pathway, and it is possible that LRFN4 may operate via a comparable mechanism. However, the precise molecular mechanisms underlying these effects remain unclear. It is plausible that LRFN4 modulates key signaling cascades involved in cell growth, survival, and motility, such as the MAPK, Wnt/β - catenin, or TGF - β pathways, which are crucial for maintaining normal cell homeostasis. Dysregulation of these pathways by LRFN4 could shift the balance towards uncontrolled cell growth and transformation.

The significant association between LRFN4 expression and pathological stages in specific tumors, including ACC, LUAD, KIRC, LUSC, and OV, strongly indicates that LRFN4 could serve as a valuable biomarker for monitoring disease progression. However, the variable correlation between LRFN4 expression and overall survival (OS) and disease-free survival (DFS) across different cancer types underscores its complex prognostic value. In colorectal cancer, high LRFN4 expression is associated with favorable OS, while in ACC, CESC, LUAD, and LIHC, it predicts a poor prognosis. This variability can be attributed to the distinct molecular and cellular landscapes of different tumors. Each cancer type possesses unique genetic mutations, epigenetic modifications, and microenvironmental factors that can influence the function of LRFN4. For example, in tumors with high levels of certain growth factors, LRFN4 may interact with these factors in distinct ways, leading to divergent effects on cellular behavior. Although the receiver operating characteristic (ROC) analysis supports the potential of LRFN4 as a prognostic biomarker, its predictive ability is confounded by factors such as tumor heterogeneity, immune infiltration, and genetic alterations. Tumor heterogeneity can lead to variations in LRFN4 expression within a single tumor, making it difficult to accurately assess its prognostic value. Immune infiltration can modulate the tumor microenvironment, and LRFN4 may interact with immune cells in a context-dependent manner. Genetic alterations can also affect LRFN4 expression and function. Therefore, a comprehensive evaluation of these factors is essential when evaluating the prognostic significance of LRFN4. It is likely that LRFN4 interacts differentially with the TME or cellular machinery depending on the cancer type, ultimately influencing patient outcomes in various ways.

LRFN4’s upregulation in the THP-1 monocyte cell line, and its role in regulating trans endothelial migration and cell elongation of THP - 1 cells, suggests a significant involvement in monocyte and macrophage migration, processes that are closely associated with the TME (10, 11). Given the crucial role of immune cells in tumor biology, LRFN4 may profoundly influence the function and behavior of tumor-associated macrophages (TAMs) and other immune cells. It could modulate immune cell migration, infiltration, and activation through multiple mechanisms. For instance, LRFN4 might interact with chemokine receptors on immune cells, guiding their movement towards the tumor site. Once in the tumor microenvironment, it could affect the polarization of TAMs. It is hypothesized that LRFN4 promotes the conversion of TAMs into immunosuppressive M2 - type macrophages, which secrete cytokines such as interleukin-10 and transforming growth factor-β that inhibit anti-tumor immune responses. The positive correlation between LRFN4 expression and immune cell markers, including CD4+ T cells, CD8+ T cells, neutrophils, and macrophages, further validates its role in the immune microenvironment.

In liver hepatocellular carcinoma (LIHC), the positive correlation between LRFN4 and immunotherapy targets such as PDCD1 and CTLA4 implies that LRFN4 can impact the interaction between immune cells and tumor cells, potentially modulating the efficacy of immune checkpoint blockade (ICB) therapy. This interaction could involve LRFN4 - mediated regulation of immune cell activation or tumor cell evasion mechanisms. The specific correlation between LRFN4 expression and the expression of immunosuppressive checkpoint proteins (ICPs) in most tumor types, especially in LIHC, OV, and THCA, indicates that LRFN4 may be involved in immune evasion mechanisms. Tumors might exploit LRFN4 to upregulate ICPs, thereby escaping immune surveillance. This correlation can be leveraged for therapeutic purposes. In-depth research on the relationship between LRFN4 and ICPs can help identify patients more likely to benefit from ICB therapy. By targeting LRFN4, it may be possible to modulate the expression or function of ICPs, thereby enhancing the anti-tumorimmune response. However, the precise molecular mechanisms underlying this interaction remain to be elucidated. It is unclear whether LRFN4 directly binds to ICPs or operates through intermediate signaling molecules to regulate ICP expression. Further investigation is needed to clarify these mechanisms.

TMB, defined as the total number of mutations per megabase in the tumor genome’s exon region, is closely associated with tumor neoantigen production and DNA repair defects (45). MSI, a hypermutation phenotype resulting from MMR impairment, is also relevant to ICI efficacy (46). The observed correlations between LRFN4 expression and both TMB, as well as MSI, in certain cancers suggest that LRFN4 can influence the immunogenicity of tumors. TMB and MSI are important determinants of the efficacy of immune checkpoint inhibitors (ICIs), and LRFN4’s association with these factors suggests its potential role in immunotherapy. Positive or negative correlations of LRFN4 with TMB and MSI imply that LRFN4 may either enhance or suppress the generation of tumor neoantigens or affect DNA repair processes. For example, a positive correlation with TMB could suggest that LRFN4 promotes the accumulation of mutations, leading to increased neoantigen production. Conversely, a negative correlation might indicate that LRFN4 is involved in DNA repair mechanisms. Additionally, the positive correlation of LRFN4 with immune, stromal, and ESTIMATE scores suggests its role in modulating the interaction between immune and stromal cells within the TME. This modulation can contribute to the nutritional complexity and heterogeneity of the TME, ultimately affecting cancer prognosis and immunotherapy outcomes. For instance, LRFN4-mediated interactions could lead to changes the secretion of cytokines and growth factors in the TME, altering the availability of nutrients and oxygen for both tumor and immune cells.

The use of fluorescence-based mIHC technology in stomach adenocarcinoma (STAD) tissues to study LRFN4 protein expression and its correlation with clinical characteristics validates the bioinformatics findings at the protein level. The correlation with immune cell markers, especially macrophage infiltration, indicates that LRFN4 could play a role in the local immune response in gastric cancer. This aligns with its proposed role in modulating immune cell migration and infiltration. The Western Blot analysis reveals LRFN4’s dual role in regulating cell cycle progression and apoptosis. High expression levels of LRFN4 are positively correlated with increased levels of cyclin D1 and CDK4, suggesting that it may promote cell cycle progression. Conversely, high LRFN4 expression is negatively associated with decreased levels of cleaved-caspase-3, indicating that LRFN4 may inhibit apoptosis by suppressing caspase-3 activation. Moreover, the functional analysis using flow cytometry in STAD cells upon LRFN4 knockdown or overexpression provides valuable insights into its role in apoptosis and cell cycle regulation. The enhanced apoptosis following LRFN4 knockdown suggests that LRFN4 might act as an oncogene by inhibiting apoptotic pathways, thereby promoting cancer cell survival. Its role in regulating S-phase arrest indicates that LRFN4 could be involved in cell cycle control, potentially through interactions with cell cycle regulators. These findings support the potential of LRFN4 as a potential therapeutic target and underscore the importance of understanding its molecular interactions within the context of gastric cancer biology. Future studies should further investigate the molecular mechanisms by which LRFN4 influences apoptosis and cell cycle dynamics. These findings provide a foundation for future research, including a detailed exploration of the specific mechanisms of action of LRFN4 in gastric cancer and an evaluation of the therapeutic potential of LRFN4 inhibitors.

A key limitation of the current study lies in its reliance on bioinformatics analyses. Although bioinformatics provides a broad overview of LRFN4’s expression patterns and associations, it does not directly elucidate the underlying molecular mechanisms. The observed correlations and associations need to be confirmed through experimental studies to confirm their biological relevance. Additionally, the use of data primarily from the TCGA database may introduce selection bias, as the sample population might not fully represent the genetic and clinical diversity of cancer patients. This could lead to overestimation or underestimation of LRFN4’s roles in different cancer types. Future studies should include a more diverse patient cohort and sample types, as such diversification can provide valuable insights and facilitate the investigation of the molecular drivers of LRFN4 in tumor occurrence.

## 5 Conclusion

The present study strengthens the hypothesis that LRFN4 serves as a prognostic biomarker for tumors and a prospective therapeutic target. Future investigation should focus on the role of LRFN4 within the tumor immune microenvironment and its involvement in tumor responses to immunotherapy. Such studies will contribute to the development of novel treatment strategies aimed at enhancing outcomes for cancer patients.

## Data Availability

The datasets presented in this study can be found in online repositories. The names of the repository/repositories and accession number(s) can be found in the article/**Supplementary Material**.
