# Supplementary material for: A comprehensive pan-cancer analysis of LRFN4: its potential as a prognostic biomarker and therapeutic target for immunotherapy
Source: Front Immunol. 2025 May 2;16:1539076. doi: 10.3389/fimmu.2025.1539076 (PMC12081452; doi:10.3389/fimmu.2025.1539076)
Supplement: Supplementary file 1 [file DataSheet1.docx]

Supplementary Material

# Supplementary Data

# Supplementary Figures and Tables

## Supplementary Figures

**
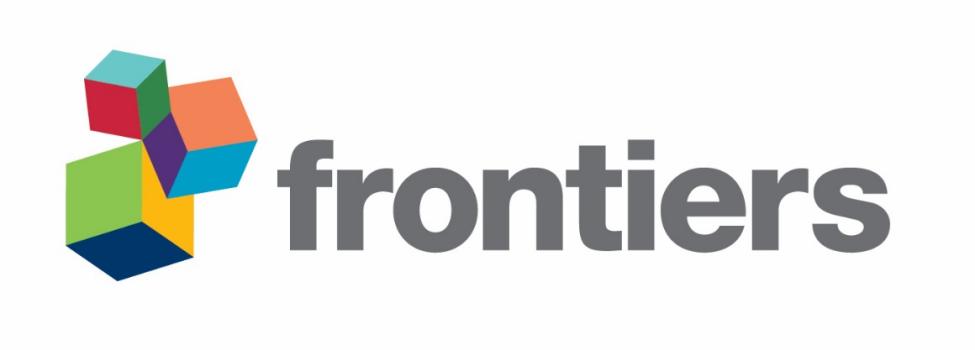
**

**Supplementary Figure 1**


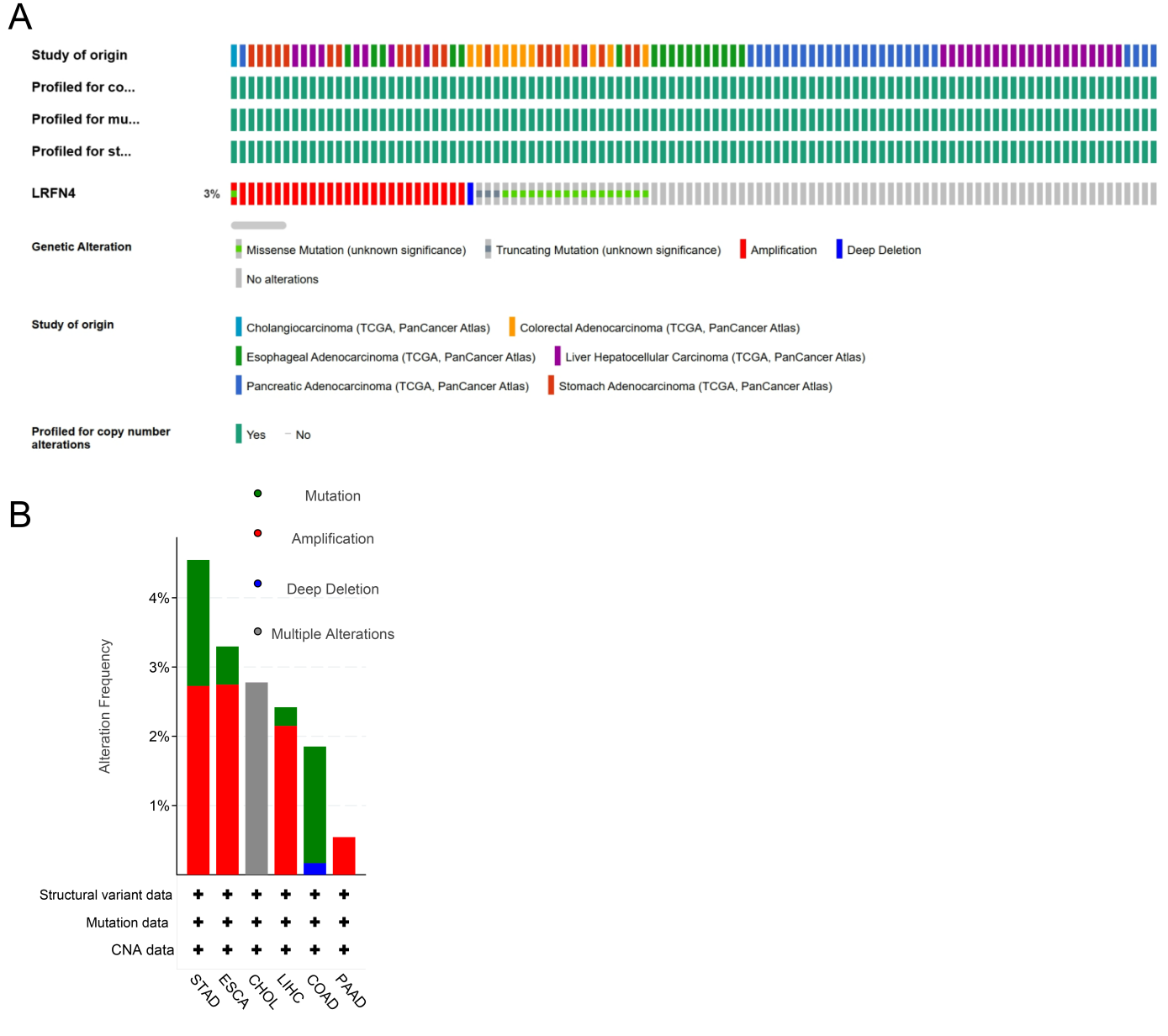


**Figure S1. Detailed genetic mutation analysis of LRFN4 in digestive system cancers.**

1. Mutation types identified in gastrointestinal cancers. (B) Mutation sites and their implications for functional domains.

**Figure S2. Validate LRFN4 gene knockdown and overexpression efficiency by qPCR and WB**

**
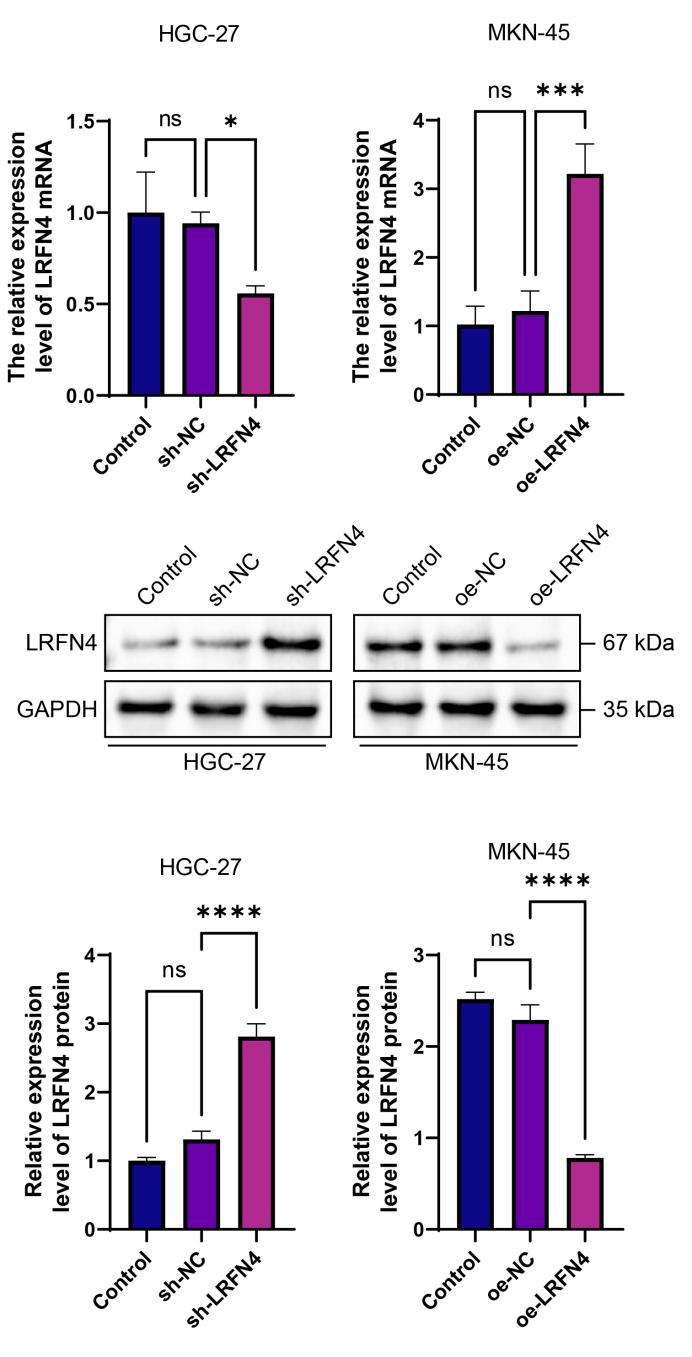
**
